# Quantitative biokinetics over a 28 day period of freshly generated, pristine, 20 nm silver nanoparticle aerosols in healthy adult rats after a single 1½-hour inhalation exposure

**DOI:** 10.1186/s12989-020-00347-1

**Published:** 2020-06-05

**Authors:** Wolfgang G. Kreyling, Uwe Holzwarth, Stephanie Hirn, Carsten Schleh, Alexander Wenk, Martin Schäffler, Nadine Haberl, Neil Gibson

**Affiliations:** 1 Institute of Epidemiology, Helmholtz Center Munich – German Research Center for Environmental Health, Ingolstaedter Landstrasse 1, 85764 Neuherberg / Munich, Germany; 2 Comprehensive Pneumology Center, Institute of Lung Biology and Disease, Helmholtz Zentrum München – German Research Center for Environmental Health, Ingolstaedter Landstrasse 1, 85764 Neuherberg / Munich, Germany; 3 European Commission, Joint Research Centre (JRC), Ispra, Italy; 4 Present address: Abteilung Gesundheit, Berufsgenossenschaft Holz und Metall, Am Knie 8, 81241 Munich, Germany; 5 Present address: Department Infrastructure, Safety, Occupational Protection, German Research Center for Environmental Health, Ingolstaedter Landstrasse 1, 85764 Neuherberg / Munich, Germany

**Keywords:** Spark ignition generated silver nanoparticle (NP) aerosols, Characterization of physicochemical NP properties, Intratracheal inhalation of freshly generated aerosols, Ionic silver released from the NP surface precipitate as low-solubility silver-salt precipitates in body fluids, Prolonged dissolution in body fluids due to low-solubility silver-salt layers on the surface of NP cores, Competing translocation of silver NP cores versus low-solubility silver-salt precipitates across the air-blood-barrier, Clearance of low-solubility silver-salt precipitates from to blood via liver and gall bladder into the small intestine, Accumulation in secondary organs and tissues, Minimal urinary excretion of any silver species

## Abstract

**Background:**

There is a steadily increasing quantity of silver nanoparticles (AgNP) produced for numerous industrial, medicinal and private purposes, leading to an increased risk of inhalation exposure for both professionals and consumers. Particle inhalation can result in inflammatory and allergic responses, and there are concerns about other negative health effects from either acute or chronic low-dose exposure.

**Results:**

To study the fate of inhaled AgNP, healthy adult rats were exposed to 1½-hour intra-tracheal inhalations of pristine ^105^Ag-radiolabeled, 20 nm AgNP aerosols (with mean doses across all rats of each exposure group of deposited NP-mass and NP-number being 13.5 ± 3.6 μg, 7.9 ± 3.2•10^11^, respectively). At five time-points (0.75 h, 4 h, 24 h, 7d, 28d) post-exposure (p.e.), a complete balance of the [^105^Ag]AgNP fate and its degradation products were quantified in organs, tissues, carcass, lavage and body fluids, including excretions.

Rapid dissolution of [^105^Ag]Ag-ions from the [^105^Ag]AgNP surface was apparent together with both fast particulate airway clearance and long-term particulate clearance from the alveolar region to the larynx. The results are compatible with evidence from the literature that the released [^105^Ag]Ag-ions precipitate rapidly to low-solubility [^105^Ag]Ag-salts in the ion-rich epithelial lining lung fluid (ELF) and blood. Based on the existing literature, the degradation products rapidly translocate across the air-blood-barrier (ABB) into the blood and are eliminated via the liver and gall-bladder into the small intestine for fecal excretion. The pathway of [^105^Ag]Ag-salt precipitates was compatible with auxiliary biokinetics studies at 24 h and 7 days after either intravenous injection or intratracheal or oral instillation of [^110m^Ag]AgNO_3_ solutions in sentinel groups of rats. However, dissolution of [^105^Ag]Ag-ions appeared not to be complete after a few hours or days but continued over two weeks p.e. This was due to the additional formation of salt layers on the [^105^Ag]AgNP surface that mediate and prolonge the dissolution process. The concurrent clearance of persistent cores of [^105^Ag]AgNP and [^105^Ag]Ag-salt precipitates results in the elimination of a fraction > 0.8 (per ILD) after one week, each particulate Ag-species accounting for about half of this. After 28 days p.e. the cleared fraction rises marginally to 0.94 while 2/3 of the remaining [^105^Ag]AgNP are retained in the lungs and 1/3 in secondary organs and tissues with an unknown partition of the Ag species involved. However, making use of our previous biokinetics studies of poorly soluble [^195^Au]AuNP of the same size and under identical experimental and exposure conditions (Kreyling et al., ACS Nano 2018), the kinetics of the ABB-translocation of [^105^Ag]Ag-salt precipitates was estimated to reach a fractional maximum of 0.12 at day 3 p.e. and became undetectable 16 days p.e. Hence, persistent cores of [^105^Ag]AgNP were cleared throughout the study period. Urinary [^105^Ag]Ag excretion is minimal, finally accumulating to 0.016.

**Conclusion:**

The biokinetics of inhaled [^105^Ag]AgNP is relatively complex since the dissolving [^105^Ag]Ag-ions (a) form salt layers on the [^105^Ag]AgNP surface which retard dissolution and (b) the [^105^Ag]Ag-ions released from the [^105^Ag]AgNP surface form poorly-soluble precipitates of [^105^Ag]Ag-salts in ELF. Therefore, hardly any [^105^Ag]Ag-ion clearance occurs from the lungs but instead [^105^Ag]AgNP and nano-sized precipitated [^105^Ag]Ag-salt are cleared via the larynx into GIT and, in addition, via blood, liver, gall bladder into GIT with one common excretional pathway via feces out of the body.

## Background

By range of applications, silver nanoparticles (AgNP) are the most frequently used nanomaterial due to their antimicrobial, cytotoxic and electrical properties. In 2014 the Nanotechnology Consumer Products Inventory (CPI) of the Woodrow Wilson International Center for Scholars listed 435 (out of 1814) products containing nano-silver [1]. Silver nanoparticles (AgNP) have been used extensively (a) in electronic products (b) as anti-microbial and anti-bacterial agents used in wound dressings, sprays, textiles, and medical devices, (c) in food storage and food packaging, (d) as textile coatings and (e) in a number of environmental applications [1–3] such as water treatment [4]. Moreover, the textile industry has started to use AgNP in different textile fabrics [2, 3]. Recently, inkjet technology has been used to produce flexible electronic circuits, using nano-sized metal particles such as Au or Ag of high electrical conductivity [2, 3, 5] uniformly dispersed in the inks. Overall 25% of the above-mentioned products apply or contain nanomaterials in a possibly inhalable way for example as sprays [1], which deserves special attention since AgNP may penetrate the air-blood barrier (ABB) and even subsequent barriers such as the placental barrier [6].

A recent review [7] summarizes the numerous ways to generate AgNP: currently, Ag-NP are being fabricated on an industrial scale utilizing physico-chemical techniques such as chemical reduction, gamma-ray radiation, microemulsion techniques, electrochemical methods, laser ablation, autoclaving, microwaving, and photochemical reduction. These methods are all effective but suffer from several limitations such as the use of toxic ingredients, high operational cost, and high energy consumption. In order to address these weaknesses, recently “green methods” to synthesize AgNP have been advanced. These methods make use of the capabilities of some microorganisms such as certain bacteria, fungi, yeasts, algae, or plant extracts to reduce and/or stabilize certain silver compounds thereby forming AgNP. Depending on the synthesis methods and the synthesis conditions, AgNP produced by green methods vary in size, shape, surface electric charge, and in other physico-chemical characteristics. Like other nanomaterials, nanosized Ag-particles are several times more reactive than the corresponding bulk particles and exhibit much more pronounced catalytic effects. However, these desired properties may also increase the toxicity of the nanoforms due to their capability to generate reactive oxygen species (ROS).

In vivo and in vitro toxicity of AgNP has been reported in numerous research papers and reviews [8–13]. The present work deals with the effects of pristine AgNP, generated by spark ignition aerosol generarion between two pure silver electrodes and immediately used for inhalation experiments with rats. Therefore, we refer mainly to investigations that refrained from applying coatings or capping agents in order to prevent AgNP agglomeration in suspension. Here we investigate rather the interactions of inhaled pristine AgNP with the biological environment, and their subsequent fate in the lungs and the entire organism of healthy rats [14–22].

Substantial evidence exists to suggest that the adverse effects induced by AgNP are predominantly mediated via Ag^+^ ions that are released from the particle surface [7, 21, 23, 24]. Therefore, and in contrast to our earlier experiments with inhaled 20 nm nanoparticle-sized aerosols of gold [25], iridium [26–28], elemental carbon [29], or titanium dioxide [30] we expect clearly observable differences in the translocation across the air-blood-barrier (ABB), the biodistribution in the entire organism and the excretion kinetics of AgNP due to the instability of AgNP which is mediated by gradual oxidation leading to Ag^+^ ion release and AgNP dissolution [31]. However, the thermodynamically possible full oxidation and dissolution of AgNP has never been observed in biological systems, and the fast, initial Ag^+^ ion release gradually slows down [32]. Therefore, these authors conclude that AgNP are protected against complete dissolution by the formation of very stable Ag_6_O octahedra on their surface when oxygen radicals penetrate the nanoparticle surface.

Furthermore, Li and co-workers [33] showed that in biological fluids containing high Cl^−^ concentrations the released Ag^+^ ions precipitate as solid AgCl (with a very low solubility constant (Ksp = 1.77 10^− 11^ mol/L)) on the AgNP. The precipitated AgCl can form both, nano-sized silver clusters in the body fluid or they can form smooth surface layers on AgNP thereby dramatically altering the AgNP morphology [31]. TEM images confirmed by X-ray diffraction showed clusters formed by partially dissolved Ag which adhere to each other and form agglomerates of irregular shape and size. The smooth surface of the adhered Ag-clusters is attributed to continued AgCl precipitation on their surface [31]. Therefore, the decrease in the soluble silver species concentration in body fluids containing chloride ions is attributed to the formation of AgCl clusters and to AgCl(s) precipitation as shells on persistent cores of AgNP [34, 35]. When exposed to human synthetic stomach fluid containing HCl and pepsin, a pronounced release of Ag^+^ ions has been reported as well as a pronounced agglomeration of the AgNP with AgCl being present on the surfaces and interfaces between the AgNP [36, 37]. Levard and coworkers [38] also reported sulfidation of AgNP surfaces since silver readily reacts with sulfide to form Ag(0)/Ag_2_S core−shell particles; i.e. elemental silver in the AgNP is oxidized to Ag^+^, which then reacts with inorganic sulfide abundantly present in biological fluids to form secondary Ag_2_S NPs or core−shell Ag:Ag_2_S particles. As the concentration ratio of sulfur to silver increases, the release of Ag^+^ ions from the AgNP decreases [3, 31].

The formation of secondary nanoparticles containing silver has been reported by Juling et al. [39] after oral delivery of 15 nm AgNP to rats as well as after intravenous injection of silver ions in the form of silver acetate. By TEM and EDX analyses of liver tissue after oral AgNP delivery, particles containing silver and sulfur in a size range of 5 to 12 nm were observed inside different types of liver cells. Small silver particles of about 6 nm were also detected at high numbers in the livers of rats even after intravenous injection of silver acetate, which shows that nano-sized Ag-clusters can be formed in vivo from Ag^+^ ions de novo [39].

Further evidence for the formation of secondary nanoparticles comes from an oral exposure study using either 20 nm AgNP or AgNO_3_ solution [40]. In this study, Ag-clusters were detected using single-particle ICPMS in organs of both groups of animals that were orally instilled with either 20 nm AgNP or with AgNO_3_ solution. Similar results were found by [31, 41] as well as in argyria patients who had ingested soluble forms of silver only [42, 43] where also selenium-rich nanoparticles were detected. The concentration of Se in living organisms is much lower than that of S, but Se binds more strongly to Ag than S and may, therefore, replace S in Ag_2_S gradually over time forming more stable Ag_2_Se nanoparticles. The capability of Ag^+^ ions to form nanoparticulate Ag_2_S and Ag_2_Se in vivo and the capability of AgNP to dissolve implies that the toxicity of Ag is subject to an interplay between various chemical processes. The formation of secondary nanoparticles and nano-sized silver salt clusters [44] from Ag^+^ ions, consisting of precipitated, poorly soluble AgCl_3_, Ag_2_S, Ag_2_Se, Ag_2_PO_4_ [44], explains well why similar effects are observed irrespective whether Ag^+^ ions or AgNP are applied [43]. This is also in line with similar organ distribution patterns of Ag found in rats after oral exposure to AgNP and to Ag acetate [40, 41].

In an early study published in 2001, Takenaka and co-workers [45] described similar clearance kinetics patterns of inhaled AgNP – generated in a manner similar to the methodology of the current study – and an AgNO_3_-solution instilled into the lungs of rats. Since at that time the authors were not aware of the subsequent work discussed above, they were not able to fully interpret the results of their study.

In order to analyze the results and deduce the biokinetics and competitive clearance pathways of AgNP after inhalation of freshly produced, pristine, radiolabeled, 20 nm-sized [^105^Ag]AgNP by healthy rats, three auxiliary biokinetics studies were additionally performed in the present study by applying [^110m^Ag]AgNO_3_-solutions, (i) by intravenous injection (IV) into blood, (ii) by intratracheal instillation (IT) into the lungs and (iii) by oral instillation (GAV) into the gastro-intestinal-tract (GIT).

This biokinetics study on inhaled 20 nm [^105^Ag]AgNP is the fifth in a series that used the same inhalation apparatus and methodology, the same strain of adult healthy rats and the same biokinetics analysis methodology as for four different, 20-nm-sized, poorly soluble radio-labeled NP materials applied in exactly the same way. This allowed us the unique possibility of comparing the in vivo fate of inhaled AgNP - which are expected to dissolve partially during the experiments – with that of the poorly soluble, more stable nanoparticles.

## Results

### Aims and rationale

The present study was designed to investigate the quantitative biokinetics of AgNP after a single 1½ -hour intratracheal inhalation exposure of ^105^Ag radio-labeled, 20 nm [^105^Ag]AgNP over a period of up to 28 days. Female, adult Wistar-Kyoto rats inhaled an [^105^Ag]AgNP aerosol freshly generated by spark ignition between two pure, proton-irradiated silver electrodes. The study design is presented in Table 1. The study is part of a series of studies comparing the biokinetics and accumulation of five different inhaled 20-nm NP-materials – AgNP (this study), IrNP [26–28], AuNP [25], elemental carbon NP [29] and TiO_2_-NP [30] – including their translocation kinetics across the air-blood-barrier (ABB) and their subsequent gradual uptake from the blood into secondary organs and tissues up to 28 days post-exposure (p.e.). In order to ensure comparability, the same strain of rats, the same inhalation technology and the same analytical methodology of the biokinetics was used for all experiments. From the previous biokinetics results, it is known that nanoparticle translocation and accumulation occurs rather rapidly during the first 24 h. Therefore, the present investigation covers early accumulation with three time-points of 0.75 h, 4 h, and 24 h, followed by two time-points after 7 days and 28 days to investigate possibly slower processes of accumulation, redistribution, and clearance of nanoparticles.

**Table 1:** Design of the [^105^Ag]AgNP biokinetics study and the auxiliary studies after intravenous injection, intratracheal instillation, and intra-oral instillation (gavage) of [^110m^Ag]AgNO_3_

Female, healthy, adult WKY rats, 8–10 weeks old		dissection time-points for biodistribution analyses						
		0.75 h/1st^a^	0.75 h/2nd^b^	4 h	24 h/1st	24 h/2nd^b^	7d	28d
[^105^Ag]AgNP Inhalation, 1½ -hour exposure	# of rats	4	4	4	4	4	4	4
Intratravenous injection of soluble [^110m^Ag]AgNO_3_	# of rats				4		4	
Intratracheal instillation of soluble [^110m^Ag]AgNO_3_	# of rats				4		4	
Oral instillation (gavage) of soluble [^110m^Ag]AgNO_3_	# of rats				4		4	

^a^ Note: rats were exsanguinated and dissected immediately after the 1.5-h exposure; “0.75 h” represents the mean time-point of the 1.5-h exposure

^b^ The measurements at 0.75 h and 24 h p.e. were repeated using a second group of rats, respectively, in order to test data reproducibility during the rapidly changing biokinetics directly after inhalation

Study design of the [^105^Ag]AgNP biokinetics study in adult rats from immediately after inhalation up to 28 days p.e. The number of rats at each dissection time-point is given. Auxiliary biokinetics studies were performed after intratracheal instillation, intravenous injection, and oral instillation (gavage) of [^110m^Ag]AgNO_3_ solutions at two dissection time points 24 h and 7 days p.e.

The added value of these studies is related to the small size of the agglomeration-controlled nanoparticles in the aerosols which were inhaled immediately after generation. In many other inhalation studies, the applied NP agglomerates were much larger than 20 nm when inhaled by the rats. Moreover, the use of radiolabeled NP in this study provides the required precision and an-easy-to-use analytical methodology to study translocation kinetics across the ABB and also to quantify minor NP accumulations in secondary organs and tissues and in excretions.

Since we have previously observed intrapulmonary relocation of NP after intratracheal inhalation of 20 nm [^195^Au]AuNP [25], [^192^Ir]IrNP [26–28] and [^48^V]TiO_2_-NP [30] into the alveolar interstitium and subsequent re-entrainment back onto the lung epithelial surface, we hypothesized that the same kinetics would not necessarily be observable for the more soluble inhaled [^105^Ag]AgNP of the same size. Moreover, we have to keep in mind that the balance of the radioactivity measurements of the ^105^Ag and of ^110m^Ag represent the applied dose of [^105^Ag]AgNP and of [^110m^Ag]AgNO_3_, respectively. However, the ^105^Ag activity in individual organs and those from feces and urine will be the sum of three contributions: (i) ^105^Ag still incorporated in residual [^105^Ag]AgNP, which may exhibit a slightly smaller size due to their partial dissolution (ii) the activity of [^105^Ag]Ag^+^ ions released from the [^105^Ag]AgNP, and (iii) the activity of [^105^Ag]Ag^+^ ions incorporated in precipitates of low solubility formed in epithelial lining fluid (ELF) and/or in blood containing high amounts of ions such as chloride, phosphate, and sulfide. These precipitates can be considered as secondary nanoparticles. However, poorly soluble [^105^Ag]Ag salts may also create surface-coatings on primarily deposited [^105^Ag]AgNP. Due to the rapidly occurring biochemical transformations of [^105^Ag]Ag^+^ ions in body fluids with their abundant presence of chloride, phosphate and sulfide ions, it is rather unlikely to find ^105^Ag activity in purely ionic form as summarized in the Background section. It should be noted, however, that from radioactivity measurements of ^105^Ag or ^110m^Ag in organs, tissues and excretions it is not possible to distinguish between Ag^+^-ions or primary or secondary nanoparticles, which is a major difference with earlier experiments using de facto unsoluble nanoparticles.

### [^105^Ag]AgNP aerosol exposure and deposition

Table 2 compiles the key characterization parameters of the [^105^Ag]AgNP aerosols used for each group of rats. These were derived from in situ measurements during inhalation exposure using a Scanning Mobility Particle Sizer (SMPS) Spectrometer and a Condensation Particle Counter (CPC) synchronized with the flight time required until the inhalation by the rats (assuring the correct measurement of the contemporary size distribution at inhalation), as well as γ-spectrometry results on a filter collecting a fraction of the aerosol in a bypass line. As described in detail in the Supplementary Information the count median diameter (CMD) and its geometric standard deviation (GSD) are obtained from the as-measured particle size spectra. In Fig. S1 of the Supplementary Information aerosol parameters during the inhalation exposures for all groups of rats are presented together with S/TEM images of the AgNP. Additionally, the experimentally determined particle size spectra were averaged and then fitted to a log-normal distribution, applying a least-squares method. The fitted log-normal distribution was extrapolated down to a particle size of 1 nm in order to overcome the operative threshold of the SMPS of 10 nm and to estimate the contributions of smaller particles.

**Table 2:** [^105^Ag]AgNP Aerosol parameters (mean ± SD; *n* = 4 of each group) during the entire intratracheal inhalation time of 1.5 h of each exposure

Group of rats	Instrument	0.75 h(1st)	0.75 h(2nd)	4 h	24 h(1st)	24 h(2nd)	7d	28d
CMD (nm), mean ± SD of 40 spectra. ^a^	SMPS^1^	21.9 ± 0.7	21.4 ± 0.6	21.7 ± 1.4	22.1 ± 0.1	23.8 ± 3.3	21.8 ± 0.9	21.4 ± 0.5
CMD (nm) of aver-aged extrapolated spectrum^b^	SMPS	20.7	20.4	20.2	20.6	20.2	20.5	22.4
Geom. Std. Dev. (GSD) mean ± SD of 30 spectra^a^	SMPS	1.42 ± 0.01	1.42 ± 0.01	1.42 ± 0.01	1.43 ± 0.02	1.45 ± 0.06	1.43 ± 0.01	1.42 ± 0.01
GSD of averaged extrapolated spectrum^b^	SMPS	1.42	1.41	1.41	1.41	1.41	1.41	1.49
Number concentration (# × 10^6^/cm^3^) of all 30 spectra ^b^	SMPS	6.5 ± 0.8	5.8 ± 0.7	5.53 ± 1.11	6.6 ± 0.6	6.7 ± 0.8	6.2 ± 0.5	6.1 ± 0.7
Median diameter (nm) of volume concentration of all 30 spectra	SMPS	31.0 ± 1.1	30.8 ± 1.1	32.8 ± 3.4	32.4 ± 1.3	32.3 ± 0.44	32.6 ± 1.1	30.9 ± 0.8
Volume concentration ^c^ (# × 10^9^ nm^3^/cm^3^)	SMPS	59.2 ± 9.6	50.9 ± 9.3	50.9 ± 2.2	66.5 ± 10.5	69.4 ± 9.1	62.7 ± 10.3	54.2 ± 8.7
Number concentration (# × 10^6^/cm^3^)	CPC	7.9 ± 0.3	8.5 ± 0.4	7.0 ± 0.5	7.7 ± 0.2	8.2 ± 0.5	11.6 ± 11.9	8.0 ± 0.3
Specific ^105^Ag aerosol activity on filter (kBq/L)^d^	γ-spectro metry	3.41	2.80	3.80	2.64	2.20	2.69	2.24
Aerosol mass conc (μg/L) from elec-trode + filter act^d^	γ-spectro metry	1.31	1.46	1.01	1.03	0.86	1.08	0.85

^1^ AIM - Instrument Manager Software of TSI Inc. version 7.2.5.0

a Mean of count median diameters (CMD) and geometric standard deviations (GSD) of all 30 SMPS spectra measured in the size range 10 nm - 420 nm

^b^ CMD and GSD of the number size spectrum averaged over all 30 SMPS spectra and mean-square fitted and extrapolated to 1-nm size; see Supplementary Information

^c^ Integral particle volume concentration (PVC) of the aerosol distribution (cm^3^/m^3^) SMPS derived

^d^ Specific [^105^Ag]AgNP aerosol activity determined from an aerosol filter sample continuously collected during each 1.5-h exposure at 0.3 L/min according to Eq. 1 of Supplementary Information

^#^ Derived [^105^Ag]AgNP aerosol mass concentration (μg/L) dividing the specific ^105^Ag aerosol activity of the filter (, kBq/L) by the specific [^105^Ag]AgNP activity concentration 2.60 kBq/μg of the Ag-electrodes used for spark-ignition aerosol generation

In order to examine the dissolution behavior of the spark ignition generated [^105^Ag]AgNP, we performed a simple in vitro test in which the NP collected on a filter during the exposure of the group of rats dissected 24 h p.e. were tightly covered with a plain sandwich filter in a filter holder and submersed in distilled water; the dissolved [^105^Ag]Ag fraction in the water was measured γ-spectrometrically after submersion times of 15 min, 1 h, 1 day and 3 days. More experimental details and the data are presented in Fig. S2 of the Supplementary Information. Basically, the [^105^Ag]AgNP used in the present study showed a similar pattern of partial oxidative dissolution previously reported by Kittler and Lonza and their co-workers [46, 47].

Intratracheal inhalation exposure allowed deep breath ventilation and avoided head airway deposition, thus leading to enhanced intrathoracic conducting airway deposition as well as alveolar deposition, with long-term alveolar retention being the dominant outcome. Parameters of aerosol inhalation and deposition are compiled in Table 3 for each group of rats. In addition, the activity fractions cleared from the lungs that can be attributed to early clearance into the gastro-intestinal-tract (GIT) and feces up to 2 days p.e. and the long-term clearance of [^105^Ag]AgNP and their degradation products formed in the retention period from 3 days up to 28 days p.e. are given for each group of rats. Note that the retained alveolar [^105^Ag]AgNP fraction at the time of dissection diminishes rapidly at days 7 and 28 p.e., due to the rapid transformation and clearance of the [^105^Ag]AgNP. With a mean rat bodyweight of 204 ± 13 g, the mean deposited AgNP mass per body weight is 125 μg•kg^− 1^.

**Table 3:** Summary of the averages (± SD) of parameters and basic results of the biokinetics study after ^[105^Ag]AgNP inhalation

Time-point after inhalation	0.75 h(1st)	0.75 h(2nd)	4 h	24 h(1st)	24 h(2nd)	7d	28d
**Inhaled aerosol volume (L)**^**1**^	37 ± 27	36 ± 27	36 ± 27	35 ± 26	37 ± 28	40 ± 30	40 ± 30
**Inhaled [**^**105**^**Ag]AgNP activity (kBq)**^**1**^	124.7 ± 3.3	101 ± 3.1	135.3 ± 7.8	118.3 ± 2	97.9 ± 5.7	112.7 ± 4.8	88.7 ± 3.5
**Deposited**^**[105**^**Ag]AgNP activity (kBq)**^**1**^	31.4 ± 9.6	29.6 ± 8.4	37.8 ± 7.1	38.7 ± 4.91	36.1 ± 8.86	34.2 ± 13.9	37.9 ± 13.6
**Deposited AgNP mass (μg)**^**1**^	12.1 ± 3.7	11.4 ± 3.2	14.6 ± 2.7	14.9 ± 1.9	13.9 ± 3.4	13.2 ± 5.3	14.6 ± 5.3
**Deposited number of AgNP (# •10**^**11**^**)**	5.78 ± 1.77	6.63 ± 1.89	5.59 ± 1.04	7.13 ± 0.91	8.45 ± 2.08	11.32 ± 4.59	10.82 ± 3.91
**Deposited fraction (inhaled)**^**1**^	0.25 ± 0.08	0.29 ± 0.09	0.28 ± 0.06	0.33 ± 0.04	0.37 ± 0.09	0.30 ± 0.11	0.43 ± 0.16
**Retained alveolar [**^**105**^**Ag]AgNP fraction (/ILD) at dissection**^**1**^	0.90 ± 0.02	0.88 ± 0.05	0.69 ± 0.01	0.55 ± 0.03	0.62 ± 0.05	0.14 ± 0.09	0.041 ± 0.02
**Fast early cleared fraction (/ILD)**^**1**^	0.07 ± 0.01	0.09 ± 0.04	0.27 ± 0.01	0.36 ± 0.03	0.30 ± 0.02	0.60 ± 0.12	0.57 ± 0.04
**Slow long-term cleared fraction (/ILD)**^**1**^						0.25 ± 0.03	0.35 ± 0.05

^1^ according to **Eqns. 1–8, 10–16** of Supplementary Information

Parameters of the intratracheal inhaled [^105^Ag]AgNP aerosol inhalation and deposition for each group of rats: inhaled aerosol volume and ^105^Ag activity, deposited ^105^Ag activity and the corresponding deposited [^105^Ag]AgNP mass and NP number, deposited ^105^Ag activity as a fraction of the inhaled ^105^Ag activity. Additionally, the retained alveolar fraction at the time of dissection, the early clearance during the first two days p.e. and the integral long-term clearance of [^105^Ag]AgNP including their secondary products from day 3–28 after inhalation is given; the latter is calculated based on fecal excretion and retention in the gastro-intestinal-tract (GIT). All fractional data are normalized to the Initial Lung Dose (ILD), i.e. the sum of ^105^Ag radioactivities of all organs and tissues including excretion

The estimated mean deposited [^105^Ag]AgNP fractions relative to the inhaled aerosol show considerable inter-subject variability (as shown by their standard deviations) indicating that the tidal volume calculated from Eq. (2) of the Supplementary Information is only a rough estimate. In addition, the deposited fractions (per inhaled [^105^Ag]AgNP activity) in line 6 of Table 3 are consistently lower (0.3–0.4 for 24 h until 28d groups of rats) than the total deposited fraction of 0.6 as calculated by the MPPD software 3.04 (see Fig. S5 of the Supplementary Information). This may have resulted from low-pressure ventilation in the plethysmograph.

### Auxiliary studies of the biokinetics after intravenous injection and intratracheal instillation of soluble [^105^Ag]AgNO_3_ salt solutions

In the auxiliary biokinetics study after IV injection of an [^110m^Ag]AgNO_3_-solution in rats, the silver was mainly cleared into the GIT and almost completely excreted in the feces with negligible urinary excretion. After IT instillation of an [^110m^Ag]AgNO_3_-solution, we observed an Ag-fraction in the GIT and feces which was much higher than expected for fast mucociliary clearance.

**Fig. 1:**
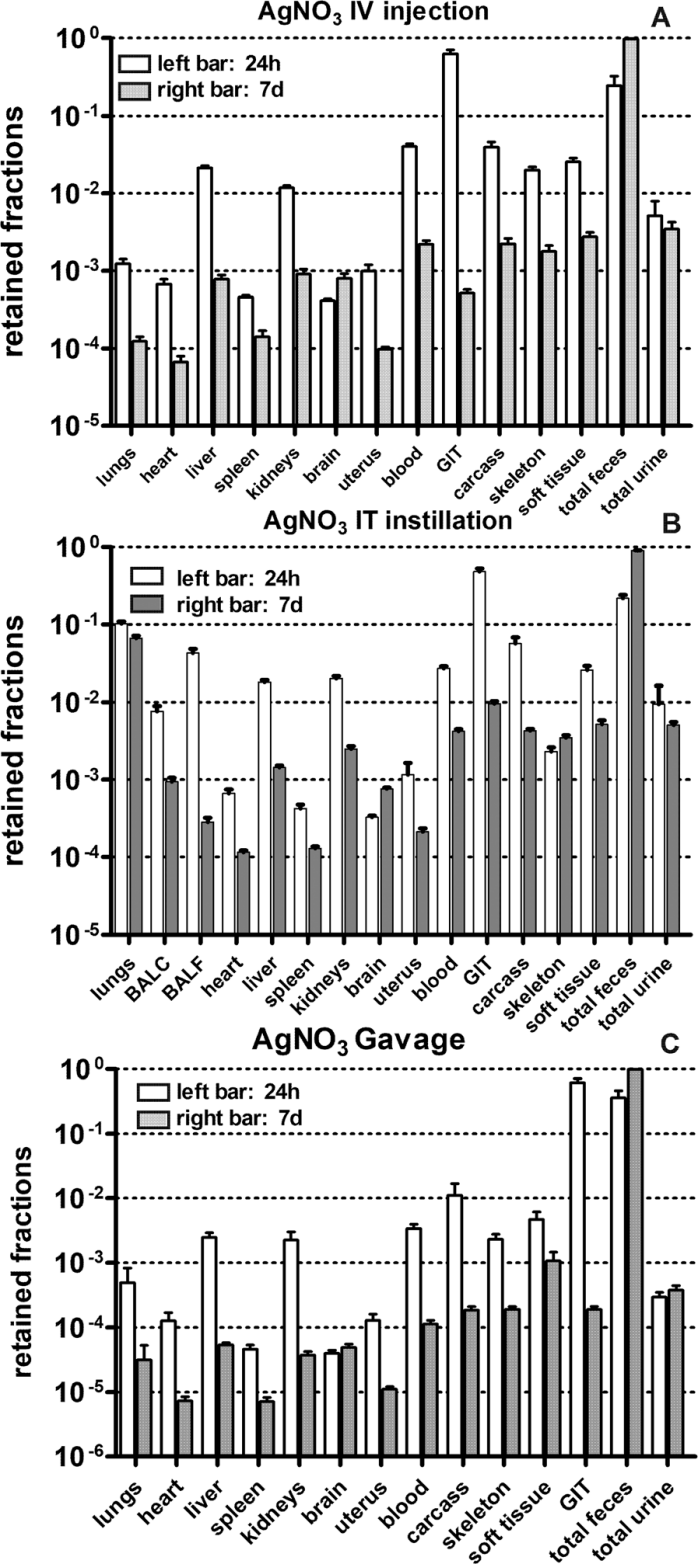
Biodistribution of [^110m^Ag]Ag after intravenous injection and intratracheal instillation of soluble [^110m^Ag]AgNO_3_ salt solutions 24 h and 7 days p.e. The graphs show completely balanced biodistributions 24 h and 7 days after intravenous injection (panel **a**) and intratracheal instillation (panel **b**) of soluble [^110m^Ag]AgNO_3_ salt solutions showing all organs, tissues and body fluids in the rat organism and total excretion via urine and feces. Data are given as mean ± SEM, *n* = 4 rats per time-point. The pattern after oral instillation is given given in panel **c**

Figure 1 shows the biodistributions of intravenously injected, (IV, **panel A**) and of intratracheally instilled (IT, **panel B**), and of intraesophageal instilled (GAV, **panel C**) [^110m^Ag]AgNO_3_ salt solutions (fully dissociated at the time of application) in four groups of rats (*n* = 4) after 24 h and 7 days, respectively. Already 24 h after IV injection (Fig. 1a), a fraction of 0.96 was eliminated from the blood circulation. Small fractions (< 0.04) were retained in secondary organs and the tissues of the remaining carcass. In contrast, predominant fractions were found in GIT (0.63) and feces (0.24). When distinguishing the GIT into its compartments - stomach, small intestine, and hindgut -, ^110m^Ag activity fractions of 0.002, 0.017 and 0.61, respectively, were found 24 h p.e. These data confirm that after IV-injection the passage into and through the small intestine is fast and almost complete within 24 h p.e. Noteworthy is the minimal urinary excretion of 0.005 24 h p.e. (and 0.0005 after GAV). After 1 week the fractions in all secondary organs and the carcass had decreased tenfold and more, but in the GIT the decrease was even 1000-fold due to almost complete fecal excretion of [^110m^Ag]Ag.

Only an [^110m^Ag]Ag fraction of about 0.14 of the intratracheally instilled material is retained in the lungs and BAL 24 h p.e. (Fig. 1b) which continues to slightly decrease during the following week to 0.07. Thus 24 h after IT instillation, a fraction of 0.86 has been translocated across the ABB into blood and fractions of 0.48 and 0.22 were found in the GIT in feces, respectively. In contrast, retained fractions in the liver, kidneys (~ 0.02 each) and carcass (0.06), and even lower fractions in other secondary organs were found, indicating rapid elimination from blood and all secondary organs and tissues into the GIT. The translocated fraction across the ABB into blood increased to 0.9 at 7 days p.e. while the fractions in all secondary organs and tissues decreased sharply. Noteworthy is the fact that the urinary excretions (< 0.01) are minimal 24 h and 7 days p.e. After gavage fractions of secondary organs and tissues fractions are even lower than after IV injection (Fig. 1c).

### Retention of [^105^Ag]AgNP and their degradation products in the lungs and BAL

**Fig. 2:**
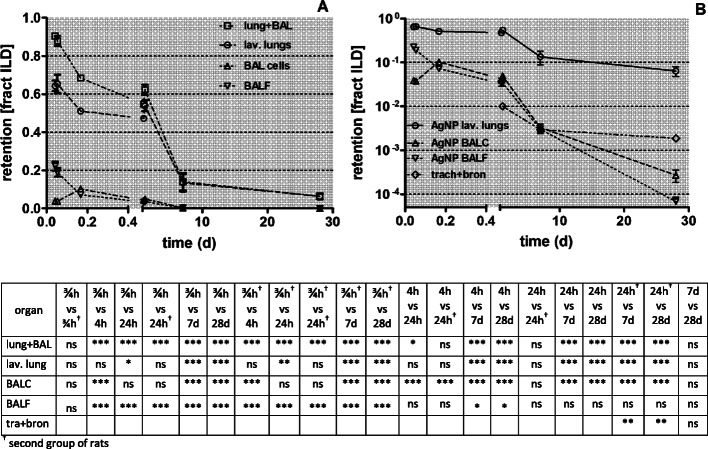
Fractional [^105^Ag]Ag retention after intratracheal inhalation of [^105^Ag]AgNP in the total lungs, the lavaged lungs and BAL between 0.75 h and 28 days p.e. In panel** a**: the retention of intratracheally inhaled [^105^Ag]AgNP and their degradation products in the total lungs between 0.75 h and 28 days are shown on a linear scale; in panel** b**: the recoveries in BALC and BALF and in trachea & bronchi are shown on a log scale. The data are given as fractions of ILD. Data are given as mean ± SEM, *n* = 4 rats/time-point. The mean ILD in mass (number) of [^105^Ag]AgNP of all five retention time points is 25.6 ± 6.9 μg (7.94 ± 3.24•10^11^ #). Data in both panels are corrected for [^105^Ag]AgNP and their degradation products retained in the residual blood volume of the lungs. Note, the independent evaluation of the measurements of two groups of rats used for each of the dissection time points at 0.75 h and 24 h p.e., are highly reproducible for each time point and do not reveal statistical differences (see the table of statistical analyses below). In the graphs data points of both groups of rats at 0.75 h and 24 h are set slightly apart from each other for easier distinction. Statistical one-way ANOVA analysis with the post-hoc Bonferroni test in between all time-points is given in the matrix below

Fractional lung retention and BAL data relative to ILD are shown in Fig. 2a and b and in Table 4. According to Fig. 1, the sharp drop of the retention fraction in Fig. 2a of 0.4 at 24 h p.e. comprises not only mucociliary clearance from the conducting airways towards the larynx but also [^105^Ag]Ag translocation across the ABB into the blood. The lung retained fraction continues to decline rapidly during the first week p.e. such that already 0.9 of the deposited [^105^Ag]Ag activity is eliminated from the lungs at 7 days. Thereafter, the lung retention decreases moderately until 28 days p.e. The rather low retentions in BAL cells and BAL fluid, both diminishing rapidly in the same fashion after 4 h p.e., indicate rapid conformational changes of the inhaled [^105^Ag]AgNP due to dissolution. These data differ strikingly from those obtained previously from poorly soluble 20 nm [^192^Ir]IrNP [28], 20 nm [^195^Au]AuNP [25] and 20 nm [^48^V]TiO_2_-NP [30] as will be considered in the Discussion section.

**Table 4:** [^105^Ag]AgNP retention in lungs and BAL, and in secondary organs and tissues over time p.e

dissection time		0.75 h(1st)	0.75 h(2nd)	4 h	24 h(1st)	24 h(2nd)	7d	28d
**organ**		**mean ± STD**	**mean ± STD**	**mean ± STD**	**mean ± STD**	**mean ± STD**	**mean ± STD**	**mean ± STD**
lungs + BAL	Corr. resid. Blood	0.9 ± 0.02	0.88 ± 0.05	0.68 ± 0.01	0.55 ± 0.03	0.62 ± 0.05	0.14 ± 0.09	0.06 ± 0.031
lungs + BAL	fract. Conc. [1/g]	0.72 ± 0.02	0.7 ± 0.04	0.75 ± 0.04	0.67 ± 0.02	0.64 ± 0.05	0.23 ± 0.06	0.11 ± 0.06
lungs+BAL(*10^3^)	mass conc. [ng/g]	16.08 ± 5.1	14.48 ± 4.59	15.17 ± 3.41	12.23 ± 2.29	12.16 ± 4.29	2.92 ± 3.12	1.16 ± 0.46
lav. Lungs	Corr. resid. Blood	0.64 ± 0.06	0.65 ± 0.1	0.51 ± 0.03	0.47 ± 0.02	0.54 ± 0.05	0.13 ± 0.09	0.06 ± 0.03
lav. Lungs	fract. Conc. [1/g]	0.56 ± 0.05	0.57 ± 0.07	0.61 ± 0.04	0.66 ± 0.03	0.61 ± 0.06	0.24 ± 0.07	0.12 ± 0.07
lav. Lungs (*10^3^)	mass conc. [μg/g]	12.71 ± 4.69	11.87 ± 4.5	12.43 ± 3	11.95 ± 1.84	11.5 ± 4.32	3.03 ± 3.28	1.25 ± 0.51
BALC(*10^− 2^)	Corr. resid. Blood	3.86 ± 0.67	3.74 ± 0.73	10.2 ± 1.09	4.18 ± 1.25	4.92 ± 0.76	0.33 ± 0.14	0.03 ± 0.02
BALC	fract. Conc. [1/g]	3.94 ± 0.69	3.97 ± 0.97	13.97 ± 1.38	6.48 ± 1.8	7.02 ± 1.27	0.82 ± 0.21	0.06 ± 0.04
BALC(*10^4^)	mass conc. [ng/g]	8.51 ± 1.6	7.98 ± 2.28	28.21 ± 6.02	11.97 ± 4.32	12.62 ± 1.04	0.9 ± 0.76	0.09 ± 0.07
BALF(*10^− 1^)	Corr. resid. Blood	2.22 ± 0.47	1.89 ± 0.45	0.72 ± 0.12	0.33 ± 0.09	0.34 ± 0.03	0.03 ± 0.012	0.001 ± 0
BALF	fract. Conc. [1/g]	2.27 ± 0.48	2.01 ± 0.57	0.98 ± 0.16	0.51 ± 0.14	0.48 ± 0.03	0.076 ± 0.025	0.002 ± 0.001
BALF(*10^4^)	mass conc. [ng/g]	4.89 ± 0.98	3.93 ± 0.66	1.96 ± 0.39	0.93 ± 0.32	0.91 ± 0.31	0.079 ± 0.058	0.002 ± 0.001
tra. + bron.(*10^− 3^)	Corr. resid. Blood					10 ± 3.1	2.93 ± 1.6	1.86 ± 1.99
tra. + bron.(*10^− 1^)	fract. Conc. [1/g]					0 ± 0	0.7 ± 0.45	0.46 ± 0.73
tra. + bron.(*10^4^)	mass conc. [ng/g]					0 ± 0	0.05 ± 0.03	0.03 ± 0.04
liver(*10^− 3^)	Corr. resid. Blood	5.31 ± 2.16	5.02 ± 1.82	12.05 ± 1.27	19.54 ± 1.95	17.37 ± 3.72	2.66 ± 0.61	0.5 ± 0.14
liver(*10^− 3^)	fract. Conc. [1/g]	0.7 ± 0.3	0.72 ± 0.3	2.39 ± 0.27	4.26 ± 0.8	3.33 ± 0.34	0.84 ± 0.14	0.16 ± 0.05
liver(*10^1^)	mass conc. [ng/g]	1.49 ± 0.58	1.45 ± 0.67	4.78 ± 0.87	7.6 ± 0.54	6.13 ± 1.41	0.84 ± 0.48	0.18 ± 0.08
liver(*10^0^)	transloc. NP fract.	0.21 ± 0.04	0.22 ± 0.11	0.3 ± 0.05	0.24 ± 0.03	0.26 ± 0.08	0.11 ± 0.01	0.03 ± 0.01
spleen(*10^− 4^)	Corr. resid. Blood	0.78 ± 0.54	3.52 ± 4	1 ± 0.33	1.69 ± 0.1	1.52 ± 0.06	0.76 ± 0.2	0.28 ± 0.13
spleen(*10^− 4^)	fract. Conc. [1/g]	1.69 ± 1.71	10.76 ± 12.52	3.72 ± 1.21	7.92 ± 1.12	3.77 ± 2.25	4.26 ± 0.54	1.11 ± 0.47
spleen(*10^0^)	mass conc. [ng/g]	4.79 ± 3.44	25.6 ± 36.22	7.3 ± 2.16	14.2 ± 0.99	7.6 ± 5.43	4.34 ± 2.65	1.41 ± 0.81
spleen(*10^− 2^)	transloc. NP fract.	0.29 ± 0.13	0.86 ± 0.82	0.25 ± 0.08	0.21 ± 0.01	0.23 ± 0.08	0.3 ± 0.02	0.15 ± 0.11
kidneys(*10^− 3^)	Corr. resid. Blood	0.39 ± 0.12	0.41 ± 0.22	1.24 ± 0.09	5.16 ± 0.94	6.66 ± 1.35	1.31 ± 0.78	0.11 ± 0.04
kidneys(*10^−3^)	fract. Conc. [1/g]	0.23 ± 0.09	0.24 ± 0.13	0.95 ± 0.13	4.6 ± 0.76	9.33 ± 8.15	1.47 ± 0.5	0.19 ± 0.09
kidneys(*10^0^)	mass conc. [ng/g]	4.71 ± 0.72	5 ± 3.51	18.81 ± 1.95	83.97 ± 19.3	152.7 ± 98.83	17.16 ± 15.66	2.03 ± 0.39
kidneys(*10^− 2^)	transloc. NP fract.	1.58 ± 0.38	1.55 ± 0.3	3.12 ± 0.71	6.29 ± 1.15	10.04 ± 3.67	4.81 ± 1.27	0.55 ± 0.16
heart (*10^− 4^)	Corr. resid. Blood	0.95 ± 0.18	7.07 ± 11.36	2.48 ± 0.52	4.14 ± 0.35	5.07 ± 0.62	0.9 ± 0.53	0.05 ± 0.01
heart (*10^−4^)	fract. Conc. [1/g]	1.32 ± 0.3	9.22 ± 14.74	4.85 ± 1.02	9.43 ± 0.91	9.59 ± 1.8	2.93 ± 1.88	0.16 ± 0.04
heart (*10^0^)	mass conc. [ng/g]	2.98 ± 1.25	25.2 ± 44.1	9.53 ± 0.85	17.03 ± 1.83	17.87 ± 6.3	2.62 ± 1.33	0.21 ± 0.12
heart (*10^− 2^)	transloc. NP fract.	0.41 ± 0.15	1.91 ± 2.45	0.63 ± 0.2	0.5 ± 0.04	0.76 ± 0.24	0.39 ± 0.29	0.03 ± 0.01
brain(*10^−4^)	Corr. resid. Blood	0.41 ± 0.56	0.46 ± 0.37	0.26 ± 0.08	1.62 ± 0.22	1.09 ± 0.07	3.43 ± 0.74	3.64 ± 0.32
brain(*10^− 4^)	fract. Conc. [1/g]	0.22 ± 0.29	0.28 ± 0.23	0.2 ± 0.06	1.39 ± 0.2	0.89 ± 0.1	6.64 ± 4.7	4.65 ± 0.3
brain(*10^0^)	mass conc. [ng/g]	0.37 ± 0.35	0.55 ± 0.46	0.41 ± 0.16	2.5 ± 0.19	1.63 ± 0.35	5.86 ± 3.21	5.68 ± 2.41
brain(*10^− 2^)	transloc. NP fract.	0.17 ± 0.22	0.34 ± 0.36	0.07 ± 0.03	0.2 ± 0.03	0.16 ± 0.05	1.42 ± 0.43	1.88 ± 0.44
uterus(*10^− 4^)	Corr. resid. Blood	0.77 ± 0.86	3.79 ± 4.33	0.66 ± 0.67	0.84 ± 0.99	1.43 ± 2.01	0.81 ± 0.62	0.07 ± 0.02
uterus(*10^−4^)	fract. Conc. [1/g]	1.45 ± 1.62	7.54 ± 8.53	1.79 ± 1.85	2.5 ± 2.96	3.59 ± 5.04	3.27 ± 2.1	0.29 ± 0.11
uterus(*10^0^)	mass conc. [ng/g]	3.53 ± 3.94	17.2 ± 23.1	4.07 ± 4.3	4.87 ± 5.64	5.55 ± 6.88	4.07 ± 4.21	0.32 ± 0.12
uterus(*10^−2^)	transloc. NP fract.	0.3 ± 0.34	0.89 ± 0.93	0.17 ± 0.18	0.1 ± 0.12	0.17 ± 0.2	0.3 ± 0.19	0.04 ± 0.02
carcass(*10^− 2^)	Corr. resid. Blood	18.35 ± 4.26	21.2 ± 16.0	26.22 ± 8.23	51.01 ± 4.7	41.58 ± 24.65	13.23 ± 2.73	2.78 ± 0.14
carcass(*10^− 2^)	fract. Conc. [1/g]	0.12 ± 0.03	0.14 ± 0.11	0.23 ± 0.07	0.53 ± 0.05	0.33 ± 0.18	0.2 ± 0.03	0.04 ± 0
carcass(*10^1^)	mass conc. [ng/g]	0.27 ± 0.07	0.3 ± 0.26	0.47 ± 0.18	0.97 ± 0.18	0.56 ± 0.17	0.2 ± 0.12	0.04 ± 0.02
carcass(*10^0^)	transloc. NP fract.	0.74 ± 0.03	0.69 ± 0.1	0.63 ± 0.07	0.62 ± 0.04	0.55 ± 0.15	0.53 ± 0.06	0.14 ± 0.04
blood(*10^− 3^)	Corr. resid. Blood	1.55 ± 0.21	1.85 ± 1.34	4.59 ± 1.02	16.57 ± 1.64	12.47 ± 0.82	3.11 ± 0.57	0.24 ± 0.09
blood(*10^− 3^)	fract. Conc. [1/g]	0.12 ± 0.02	0.15 ± 0.12	0.5 ± 0.1	2.13 ± 0.33	1.3 ± 0.05	0.58 ± 0.08	0.04 ± 0.01
blood(*10^− 3^)	mass conc. [ng/g]	0.27 ± 0.07	0.28 ± 0.15	0.99 ± 0.14	3.8 ± 0.07	2.4 ± 0.57	0.58 ± 0.34	0.05 ± 0.03
blood(*10^0^)	transloc. NP fract.	0.06 ± 0.02	0.07 ± 0.03	0.11 ± 0.03	0.2 ± 0.02	0.19 ± 0.06	0.13 ± 0.02	0.01 ± 0
transloc.(* 10^− 2^)	Corr. resid. Blood	2.51 ± 0.64	2.91 ± 1.96	4.12 ± 0.86	8.2 ± 0.24	7.2 ± 2.47	2.56 ± 0.85	2.01 ± 0.43
transloc.(* 10^− 3^)	fract. Conc. [1/g]	0.15 ± 0.04	0.18 ± 0.13	0.32 ± 0.07	0.76 ± 0.04	0.52 ± 0.16	0.33 ± 0.02	0.23 ± 0.05
transloc.(* 10^1^)	mass conc. [ng/g]	0.33 ± 0.09	0.37 ± 0.31	0.66 ± 0.19	1.38 ± 0.18	0.91 ± 0.06	0.36 ± 0.269	0.29 ± 0.136
2nd org. (*10^− 2^)	Corr. resid. Blood	0.6 ± 0.23	0.69 ± 0.35	1.37 ± 0.14	2.55 ± 0.2	2.49 ± 0.49	0.46 ± 0.14	0.1 ± 0.02
2nd org. (*10^− 3^)	fract. Conc. [1/g]	0.37 ± 0.2	0.45 ± 0.25	1.14 ± 0.12	1.47 ± 0.24	1.59 ± 0.23	0.37 ± 0.12	0.15 ± 0.04
2nd org. (*10^1^)	mass conc. [ng/g]	0.78 ± 0.38	0.98 ± 0.82	2.29 ± 0.49	2.66 ± 0.47	2.92 ± 0.66	0.4 ± 0.31	0.17 ± 0.07
2nd org. (*10^0^)	transloc. NP fract.	0.23 ± 0.04	0.27 ± 0.09	0.34 ± 0.06	0.31 ± 0.03	0.37 ± 0.12	0.18 ± 0.01	0.05 ± 0.02

[^105^Ag]AgNP retention in lungs including broncho-alveolar-lavage (BAL) data and in secondary organs and tissues at 0.75 h, 4 h, 24 h, 7d and 28d after intratracheal inhalation; note, for 0.75 and 24 h p.e. two groups of rats were dissected for data repeatability and statistical control. The data are presented as retained [^105^Ag]Ag - activity fractions normalized to the ILD of [^105^Ag]AgNP and corrected for ^105^Ag in the residual blood in each organ (first line of a given organ). In the following two lines for each organ, the [^105^Ag]Ag -activity data were converted into [^105^Ag]AgNP concentrations per mass of organ or tissue, given as ILD fraction per gram and in NP mass ng·g^− 1^. In the fourth line of each secondary organ or tissue, fractions are normalized to the total of the [^105^Ag]Ag activity, which had crossed the ABB (transloc. NP fract., see Eqns. 23 and 24 of Supplementary Information). Mean ± SEM of *n* = 4 rats/time point.

In the caption of Fig. 2, the matrix of significances is given. This indicates highly significant changes between the early biodistributions determined within the first 24 h p.e. and the late biodistributions obtained 7 days and 28 days p.e. It is noteworthy that the independent analyses of two sets of rats 0.75 h p.e. and 24 h p.e., respectively, show no significant differences, emphasizing the high reproducibility between the two groups of rats of each time point (see also Table 4.)

### Long-term [^105^Ag]AgNP clearance

**Fig. 3:**
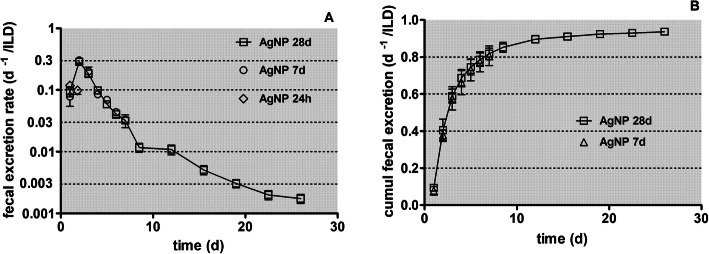
Daily fecal excretion after intratracheal inhalation of [^105^Ag]AgNP. Panel** a**: Fecal excretion rates of [^105^Ag]Ag activity after intratracheal inhalation of [^105^Ag]AgNP per lung dose (ILD); y-axis log scale. Data of the groups of rats dissected after 24 h, 7 days and 28 days are presented. For the 28 days group, integral fecal samples over 3–4 days are divided by the number of sampling days and associated with the mean day of the sample interval. Panel** b**: cumulative fecal excretion of [^105^Ag]Ag activity per ILD (y-axis lin scale). Mean ± SEM, *n* = 4 rats per time point

The daily fecal excretion per ILD is shown in Fig. 3a and the cumulative fecal excretion in Fig. 3b, based on the data retrieved from the two groups of rats dissected at 7 and 28 days p.e.

**Fig. 4:**
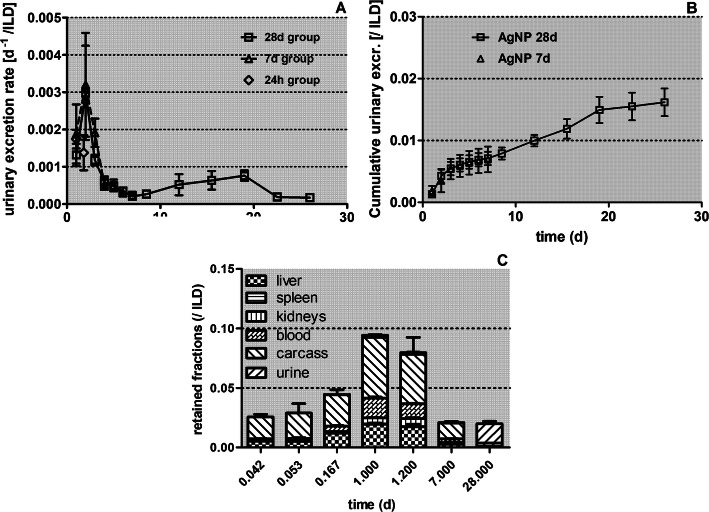
Daily urinary excreted fractions of [^105^Ag]Ag -activity after intratracheal inhalation of [^105^Ag]AgNP derived from the dissection time points 7 and 28 days p.e. Data are given as fractions of ILD. Panel** a** shows the daily excreted fractions of [^105^Ag]Ag activity; panel** b** shows the cumulative kinetics. For the 28 days group, the urine samples were collected over 3–4 days and the measured values were divided by the number of sampling days and associated with the mean day of the sample interval. Mean ± SEM, *n* = 4 rats per time point. Panel** c**: [^105^Ag]Ag activity fraction translocated across the ABB presented as stacked columns with contributions from major secondary organs, the carcass, and cumulative urine normalized to ILD of [^105^Ag]AgNP. Translocated [^105^Ag]Ag activity accumulated predominantly in the carcass and liver within the first 24 h p.e., the fraction excreted via urine increases rapidly from 7 to 28 days p.e. Mean ± SEM, *n* = 4 rats per time point

The observed cumulative fecal excretion of a fraction of more than 0.8 of the [^105^Ag]Ag activity deposited in the lungs as [^105^Ag]AgNP after the first week p.e. indicates that fecal excretion not only comprised the clearance of [^105^Ag]AgNP to the larynx but also the degradation products of the nanoparticles. The auxiliary biokinetics studies after intratracheal instillation of soluble Ag^+^ solutions (see Fig. 1) suggest that the nano-sized precipitates formed in the alveolar region may translocate across the ABB into the blood followed by rapid hepato-biliary transport into the duodenum of the GIT. Therefore, the dissolution of [^105^Ag]Ag^+^ from [^105^Ag]AgNP is only a transient process prior to the formation of nano-sized [^105^Ag]Ag-salt precipitates of low solubility in ion-rich body fluids such as ELF and blood. This is in line with the observed negligible urinary excretion (see below Fig. 4). However, our biokinetic data cannot distinguish between MCC of [^105^Ag]AgNP and precipitates such as [^105^Ag]AgCl during the first 24 h p.e. and either between the long-term clearance of persistent cores of [^105^Ag]AgNP and secondary [^105^Ag]AgCl precipitates formed in the lung periphery which also undergo long-term clearance to the larynx.

We also cannot distinguish between particulate transport from the lungs to the larynx and the GIT versus the translocation across the ABB into blood and GIT.

In Fig. 4a and b the daily urinary excretion is plotted, derived from the data retrieved from the rats of the 7-days- and the 28-day groups. These rates remain low but reach a maximum 2 days p.e of 0.006 d^− 1^ and drop sharply thereafter to around 0.001 d^− 1^. It remains speculative whether these fractions comprise soluble [^105^Ag]Ag^+^ ions and/or nano-sized [^105^Ag]AgCl_3_ precipitates of a small enough size (6–8 nm) to pass kidney clearance into the urine. Interestingly, after the first week p.e. urinary rates increase again and show a second maximum of 0.002 d^− 1^ at 18 days p.e.

In Figs. 4c the fraction of [^105^Ag]AgNP and their degradation products that were translocated across the ABB are presented as stacked columns with accumulation in major secondary organs, the carcass, and cumulative urine. The urinary fractions are low and become only visible on the linear scale 28 days p.e. The carcass shows the highest retained fractions at all time points followed by the liver and blood.

### Biokinetics of translocated [^105^Ag]AgNP into secondary organs and tissues

In Table 4 the retention of [^105^Ag]AgNP and/or [^105^Ag]Ag-salt precipitates in the lungs and in all secondary organs and tissues are presented as mean ± SEM for all dissection time points (note, the analyses at 0.75 h and 24 h p.e. were repeated in a second group of rats for each time point; in order to present the results separately, the groups have been labeled as 0.75 h/1st, 0.75 h/2nd and 24 h/1st, 24 h/2nd). The [^105^Ag]Ag -activity data are corrected for the activity in the residual blood volume of the organs and tissues and given as fractions and mass concentrations normalized to ILD, which is the total deposited lung dose applying the mathematical procedure described in the Supplementary Information. No number concentration data are provided due to the fact that the rapid transformation of the deposited [^105^Ag]AgNP starts to occur immediately after deposition due to dissolution and subsequent precipitation. However, the number of [^105^Ag]AgNP total deposited in the respiratory tract is given in Table 3.

**Fig. 5:**
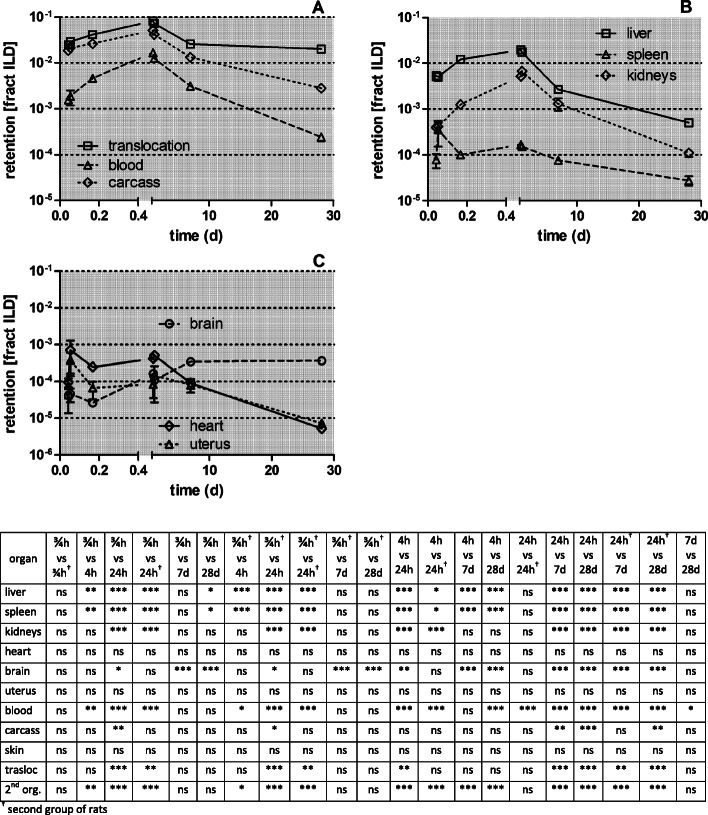
Retained [^105^Ag]Ag activity fractions after intratracheal inhalation of [^105^Ag]AgNP in organs and tissues investigated up to 28 days p.e. Panel** a**: total translocation, blood and carcass;** b**: liver, spleen, and kidneys; Panel** c**: heart, brain, and uterus;. The vertical axis of all four panels is given on a log scale. The mean ILD in mass (number) of [^105^Ag]AgNP of all five retention time points is 25.6 ± 6.9 μg (7.94 ± 3.24•10^11^ #). [^105^Ag]Ag activity retention is given as fractions of the initial lung dose (ILD) corrected for [^105^Ag]Ag in the residual blood. Data in all panels correspond to the first line (corr. Resid. blood) of each organ or tissue in Table 4. Mean ± SEM, *n* = 4 rats per time-point. Data points of both groups of rats at 0.75 and 24 h are set slightly aside from each other for easier distinction of the highly reproducible data obtained. Statistical one-way ANOVA analysis with the post-hoc Bonferroni test in between all time-points are given in the matrix below

Figure 5 displays the retained [^105^Ag]Ag -activity fractions (per ILD) of each organ or tissue corrected for residual [^105^Ag]Ag blood activity (according to the 1st line in Table 4 of each organ or tissue). Immediately after inhalation a rapid translocation into the blood is observed followed by fast uptake in the carcass, which declines until 28 days p.e. by two orders of magnitude. The increasing [^105^Ag]Ag blood activity (Fig. 5a) leads to steep increases in kidneys and moderate increases in the liver and brain. In the heart and uterus the [^105^Ag]Ag activities remain constant over the first 24 h p.e. after which they decline more than tenfold until 28 days p.e. (Fig. 5b and c). The [^105^Ag]Ag activity percentages in liver and kidneys reach about 1% of [^105^Ag]AgNP ILD while those in the heart and uterus are smaller than 0.1% of ILD. The [^105^Ag]Ag activities in the carcass reach even higher values (4%) 24 h p.e. which is more than the sum of all secondary organs. The total fraction of [^105^Ag]Ag activity translocated across the ABB reaches nearly 0.1 of ILD 28 days p.e. (Fig. 5d) which is tenfold more than the ABB-translocation of same-sized [^195^Au]AuNP after intratracheal inhalation [25].

### [^105^Ag]AgNP concentration per mass of organ or tissues (1/g) as fractions of the initial lung dose (ILD)

**Fig. 6:**
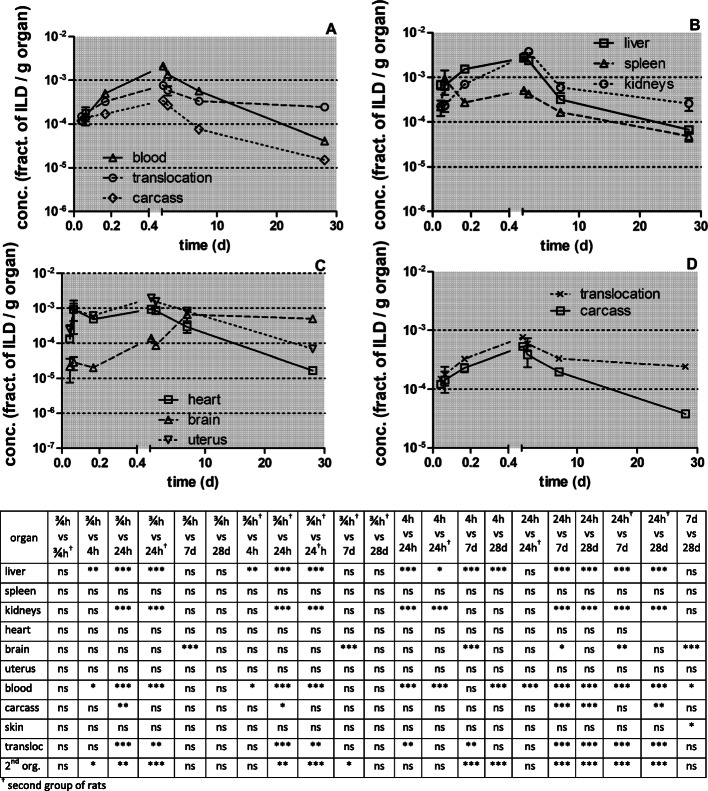
Kinetics of [^105^Ag]Ag -activity concentrations per weight of organ or tissue after intratracheal inhalation of [^105^Ag]AgNP. Panel** a**: total translocation and blood, Panel** b**: liver, spleen, and kidneys, Panel** c**: heart, uterus, and brain, Panel** d**: carcass and translocation. The mean ILD in mass (number) of [^105^Ag]AgNP for all five retention time points is 25.6 ± 6.9 μg (7.94 ± 3.24•10^11^ #). Data are corrected for [^105^Ag]Ag activity retained in the residual blood volume of organs and tissues and correspond to the second line of each organ in Table 4; data are presented as mean ± SEM; *n* = 4 rats per time point. Data points for both groups of rats at 0.75 and 24 h are set slightly aside from each other for easier distinction of the highly reproducible data obtained. Statistical one-way ANOVA analysis with the post-hoc Bonferroni test in between all time-points are given in the matrix below

In Fig. 6 the [^105^Ag]Ag activity fractions are normalized to the weight of organ or tissue. Immediately after inhalation the [^105^Ag]Ag activity concentration fractions (per organ weight) in blood and in most secondary organs (except the brain) start at about 10^− 3^ to 10^− 4^ g^− 1^ (Figs. 6a-c and Table 4) indicating rapid and uniform uptake from blood and accumulation in organs and tissues. Thereafter, Fig. 6a shows a tenfold increase of the concentration in blood at 4 h p.e. followed by a continuous decline by two orders of magnitude by 28 days p.e., which results in an approximately fivefold lower concentration than initially determined 0.75 h p.e. The initially steep increase was also found in liver, kidneys, heart, and uterus indicating rapid uptake by the MPS cells of these secondary organs when the blood concentration is still high. The decrease of the concentration between 24 h p.e. and 28 days p.e. is similar in the aforementioned organs as in the blood. The spleen shows a different behavior characterized by a gradual tenfold decrease over 28 days p.e. The [^105^Ag]Ag concentration pattern in the carcass (Fig. 6d and Table 4) follows basically the concentration pattern of blood but with lower accumulation and clearance rates.

**Fig. 7:**
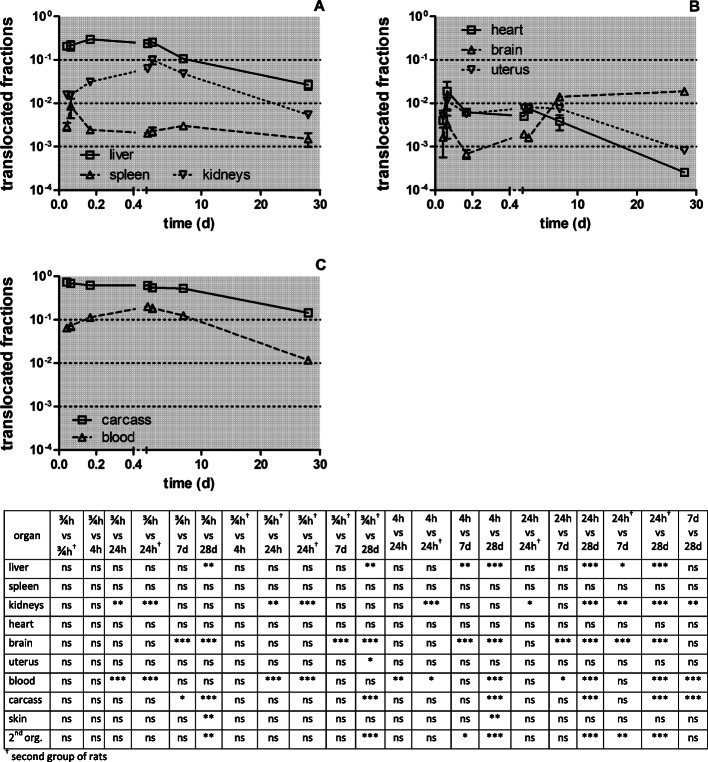
[^105^Ag]Ag activity accumulation in secondary organs and tissues between0.75 h and 28 days after intratracheal inhalation of [^105^Ag]AgNP normalized to the total ^105^Ag activity translocated across the ABB. Panel** a**: liver spleen and kidneys; Panel** b**: heart, brain, uterus; Panel** c**: carcass, blood. The data are corrected for [^105^Ag]Ag activity retained in the residual blood volume of organs and tissues. The mean ILD in mass (number) of [^105^Ag]AgNP of all five retention time points is 25.6 ± 6.9 μg (7.94 ± 3.24•10^11^ #). Mean ± SEM, *n* = 4 rats per time point. Data points for both groups of rats at 0.75 and 24 h are set slightly apart from each other for easier distinction of the highly reproducible data obtained. The table below shows the statistical one-way ANOVA analysis with the post-hoc Bonferroni test between all time-points

In Fig. 7 the [^105^Ag]Ag -activity retention in secondary organs and tissues is presented from 0.75 h to 28 days after intratracheal inhalation of [^105^Ag]AgNP normalized to the total translocated [^105^Ag]Ag activity across the ABB. The most striking feature is the rapidly accumulated and retained fraction of 0.6 of the ^105^Ag activity translocated across the ABB (in form of [^105^Ag]AgNP and/or particulate [^105^Ag]Ag-salt precipitates) in the carcass during the first 24 h p.e. At 7 days p.e. a fraction of 0.5 of the translocated [^105^Ag]Ag activity is still present in the carcass. However, 28 days p.e. the translocated fraction in the carcass declined to 0.14. Also the accumulated and retained liver fraction reaches 0.2–0.3 during the first 24 h p.e. but declines within 28 days p.e. gradually to 0.03. Kidney accumulation rises tenfold within 24 h p.e. reaching a maximal fraction of about 0.1, and declines in parallel to that in the liver after 24 h p.e. During the first week about 0.1 of the [^105^Ag]Ag -activity (most likely in form of [^105^Ag]AgNP and/or secondary particulate [^105^Ag]Ag-salt precipitates) is translocated across the ABB and remains circulating in the blood indicating that there is an ongoing interaction between the blood and the organs, mainly with liver and kidneys, but as well as with the carcass and other secondary organs. During the period from 7 days to 28 days p.e., the [^105^Ag]Ag activity declines in these organs and the carcass and is mirrored by the parallel decline of the [^105^Ag]Ag activity in the blood. In contrast, much less of the translocated ^105^Ag activity fraction is retained in spleen, heart, brain, and uterus ranging between 0.001 and 0.01.

**Fig. 8:**
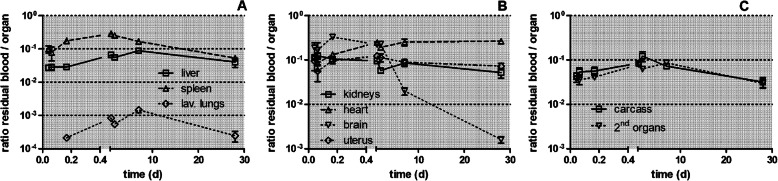
Ratio ${RB}_{\mathrm{organ},\kern0.5em \mathrm{i}}^{\mathrm{blood}\kern0.5em \mathrm{correction}}$ of the [^105^Ag]Ag activity in the residual blood over the measured organ or tissue activity. Panel** a**: lavaged lungs, liver, spleen; Panel** b**: kidneys, heart, brain, uterus; Panel** c**: carcass, secondary organs. Mean ± SEM, *n* = 4 rats per time point

In Fig. 8 the ratios of the [^105^Ag]Ag activity in the residual blood, remaining after exsanguination at the time of dissection, with respect to the total [^105^Ag]Ag activity measured in each organ or tissue are presented. Residual blood volumes are taken from Oeff and König [48] and the derivation is described in Eqns. 4–8 of the Supplementary Information. In most secondary organs about one-tenth of the [^105^Ag]Ag activity can be attributed to the residual blood volume. This ratio remains rather constant throughout the 28 days observation period (see Fig. 8a and b). For the brain, the residual blood contribution is about one-tenth during the first 24 h after which it drops sharply by two orders of magnitude. In Fig. 8c the residual blood content of the tissues of the carcass is almost identical to those of all secondary organs. In contrast, residual blood contributes minimally and rather constant over time to the lung activity indicating that in the lungs [^105^Ag]AgNP and/or particulate [^105^Ag]Ag-salt precipitates dominate the retention throughout the 28 days observation period.

## Discussion

### Previous scientific results

In this section, we will compare our experimental results after the inhalation of 20 nm [^105^Ag]AgNP with those after the inhalation of poorly soluble, 20 nm [^192^Ir]IrNP [28], [^198^Au]AuNP [25] and [^48^V]TiO_2_NP [30]. Moreover, by applying the findings of the numerous publications on the mechanisms and transformations of various silver species in vivo *-* as presented in the Background section – this will allow us to gain new insights and a better understanding of the complex biokinetics fate of inhaled 20 nm [^105^Ag]AgNP and their degradation products. For the convenience of the reader the essentials of the Background section are briefly summarized here:

- The partial [^105^Ag]AgNP dissolution that we discuss is in agreement with previous studies [47] on commercially available AgNP; it results from oxidative dissolution and depends on several parameters such as temperature, Ag-ion concentration, and oxygen availability as shown earlier [46].
- The literature presented in the Background section on the release of [^105^Ag]Ag^+^ ions from the surfaces of [^105^Ag]AgNP retained in the lungs [7, 21, 23, 24], indicate a *“rich set of biochemical transformations occurring with Ag-NP in biological media, including accelerated oxidative dissolution, thiol binding and exchange to secondary zero-valent Ag-NP”* as emphasized by [31]*.*
- The literature summarized in the Background section provides evidence that the release of [^105^Ag]Ag^+^ ions from the surfaces of [^105^Ag]AgNP immediately forms [^105^Ag]Ag-salt molecules in the abundant presence of Cl^−^, S^2−^, PO_4_^2−^ and Se^2−^ ions of the surrounding body fluids including ELF; this causes the precipitation of poorly soluble, [^105^Ag]Ag-salt clusters in the size range between 1 and 10 nm [23, 24, 34, 35, 39].
- The literature discussed in the Background section notes that the fast, initial Ag^+^ ion release gradually slows down [32] due to poorly soluble Ag-salt layers which are formed on the surface of the remaining [^105^Ag]AgNP [31, 38, 40].

**Fig. 9:**
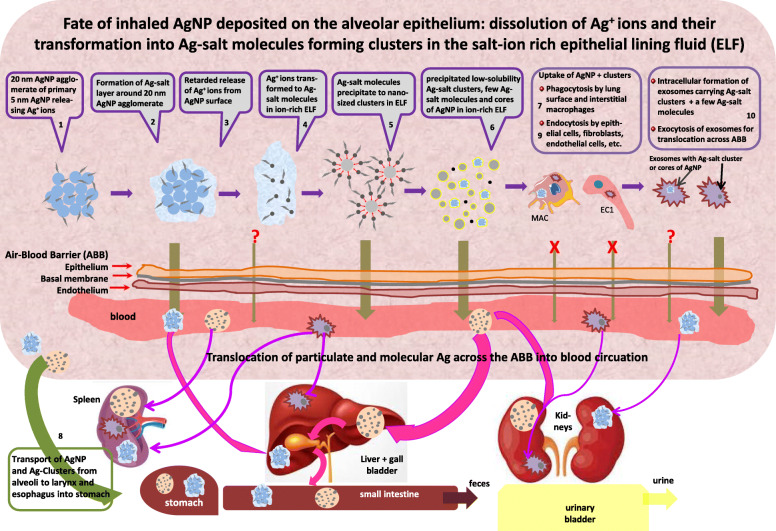
Graphical representation of the complex biokinetics after the inhalation of 20 nm [^105^Ag]AgNP. In step 1 freshly deposited [^105^Ag]AgNP start dissolving thereby releasing Ag + ions from their surface. In step 2 a fraction of the ions form layers of Ag-salt molecules around the [^105^Ag]AgNP which retards the further release of Ag^+^ ions from the NP surface (step 3). In step 4 the rest of the Ag + ions form [^105^Ag]Ag-salt molecules of low solubility in the alveolar ELF which is rich in Cl^−^, S^2−^, PO_4_^2−^ and Se^2−^ ions. Due to the high concentration of the [^105^Ag]Ag-salt molecules, they precipitate to nano-sized clusters (step 5). The [^105^Ag]Ag-salt clusters scavenge most of the [^105^Ag]Ag-salt molecules (step 6). Both the cores of [^105^Ag]AgNP and the [^105^Ag]Ag-salt clusters are phagocytized by lung surface macrophages (step 7) which will gradually transport them to the distal end of the ciliated airways for mucociliary transport to the larynx where they are swallowed into the GIT (step 8). Alternatively both particulate species may be endocytosed by cells of the alveolar epithelium (e.g. epithelial type 1 + 2 cells, fibroblasts et.) which may exocytose them in exosomes for translocation across the ABB (step 9). Translocation across the ABB of both particulate species may also occur directly from the ELF as indicated by the arrows of translocation. Hence this series of steps highlights the fate of [^105^Ag]AgNP and their degradation products, which results in the clearance of two slowly dissolving particle species – persistent cores of [^105^Ag]AgNP and clusters of low solubility [^105^Ag]Ag-salt. Once arrived in the blood both particulate species may accumulate in secondary organs and tissues as indicated schematically by liver, spleen, and kidneys and discussed below. Note that we focus here on the alveolar epithelium due to our interest in long-term particle clearance. We hypothesize that steps 1 to 6 are similarly occurring in the airway epithelium leading predominantly to mucociliary clearance

The complex biokinetics of inhaled [^105^Ag]AgNP leads to the clearance of two slowly dissolving particle species – [^105^Ag]AgNP and clusters of [^105^Ag]Ag-salt. This is schematically illustrated in Fig. 9 summarizing dissolution, transformation, and precipitation of [^105^Ag]AgNP in several consecutive steps.

As indicated in the schematics (Fig. 9) the Ag-salt layers around In addition, translocation across the ABB may be mediated by naturally occurring exosomal nanovesicles (ENV) initially synthesized in the endosomal compartment of many eukaryotic cells including macrophages and cells of the lung epithelium. When ENV fuse with the inner cell surface they are released extracellularly. They are considered to serve “as a mechanism to discharge unwanted material from the cells, but they also could form the basis of an efficient cell-cell communication mechanism” [49]. For example, when 20 nm AuNP were applied to cultured primary human macrophages the AuNP were rapidly taken up intracellularly and released within ENV [50]. Moreover, very recently ENV received much attention as natural, non-cytotoxic, nanotherapeutic carriers for specific cell targeting [51].

### Comparison of 24 h excretions after IV injection, IT instillation and GAVage of soluble ions of ^192^Ir, ^48^V, ^198^Au and of [^110m^Ag]Ag ions

In Fig. 1a we showed that 24 h p.e. IV injected soluble [^110m^Ag]AgNO_3_ was rapidly eliminated from the blood into the GIT and feces (fraction of 0.87) with only small fractions found in secondary organs and tissues. Even more surprising, in Fig. 1b we found that 24 h p.e. a fraction of IT instilled soluble [^110m^Ag]AgNO_3_ was also rapidly eliminated (0.70) from the lungs into the blood and further into the GIT and feces with only small fractions found in secondary organs and tissues and also in urine. Therefore, the question arises, how such a rapid elimination from the blood to the GIT is possible after IV injection of soluble [^110m^Ag]AgNO_3_, and what leads to such a rapid translocation across the ABB into the blood and further into the GIT after IT instillation of soluble [^110m^Ag]AgNO_3_ The literature cited in the Background section suggests rapid precipitation of low-solubility Ag-salts in blood and/or ELF, respectively, [31, 39, 41] which is sketched in Fig. 9. It basically excludes the presence of significant amounts of [^110m^Ag]Ag-ions in solution due to the abundance of salt-ions in blood and ELF which lead to the precipitation of nano-sized clusters.

However, nano-sized clusters smaller than 6–8 nm and/or macromolecules in blood would be subject to renal glomerular filtration and urinary excretion [52], which we did not observe. Instead, the [^110m^Ag]Ag-salt precipitates were rapidly eliminated into the GIT – most likely by liver uptake and the hepato-biliary clearance pathway. The size of those precipitates may play an important role. After IT instillation of monodisperse, triphenylphosphine surface-coated gold NP of 1.4 nm, 2.8 nm, 5 nm, 18 nm, 80 nm, and 200 nm diameter the translocated fraction across the ABB during the first 24 h p.e. declined rapidly with increasing NP size from almost 0.1 (of the initially delivered NP mass) for 1.4 nm NP by a hundred-fold decline for 200 nm sized particles [53]. Furthermore, after IV injection of the same set of gold NP resulting in predominant liver retention, we quantitated the hepato-biliary cleared fraction (HBC) of 1.4 nm gold NP to be 0.05 while the HBC of 2.8 nm gold NP was 0.008 and all larger-sized gold NP from 5 nm to 80 nm were only cleared by 0.005 during the first 24 h p.e [54]. This is in clear contrast to the predominant elimination of the nano-sized [^110m^Ag]Ag-salt clusters from the blood into the GIT. The results of the IT instilled and IV injected gold NP together with the absence of urinary [^110m^Ag]Ag excretion implies that the Ag-salt precipitates may (a) either have translocated across the ABB as extremely small particulates (a few-nanometers) and were scavenged rapidly in the liver or (b) the small Ag-salt precipitates increased their size during circulation to larger than about 10 nm (which seems not very plausible) or (c) that such extremely small Ag-salt precipitates are protected against renal filtration by unknown surface modifications of their biomolecular corona and/or (d) by exosomal mediation. However, the current literature does not provide any suitable candidate biomolecules blocking renal filtration and concurrently allowing hepatocytes to transcytose the [^110m^Ag]Ag-salt clusters into the Space of Dissé for further elimination through the gall-bladder into the small intestine. These remain urgent questions for future investigations.

In Table 5 the urinary and fecal excretion data after application of [^110m^Ag]Ag^+^ ions are compared with the corresponding data after application of soluble ^192^Ir-, ^198^Au- and ^48^V-ions. The comparison is done for the first 24 h after intratracheal instillation and intravenous injection. The GIT retention found within the first 24 h is added to the fecal excretion data in order to obtain the fractions that have de facto already been cleared from the lungs and are ‘ready’ for excretion. The data are compiled from auxiliary studies of previous inhalation investigations of poorly soluble [^192^Ir]IrNP [28], [^198^Au]AuNP [25] and [^48^V]TiO_2_NP [30].

**Table 5:** urinary and fecal excretion 24 h after either IV injection or IT instillation of solutions of ^192^Ir, ^48^V, ^198^Au, and ^110m^Ag ions

Ionic solutions	24 h excretion	Fraction of applied ions	Ratio urine/feces	Fraction of applied ions	Ratio urine/feces
		IV	IV	IT	IT
^192^Ir-ions	GIT + feces	0.007 ± 0.012		0.062 ± 0.019	
^198^Au-ions	GIT + feces	0.059 ± 0.006		0.201 ± 0.029	
^48^V-ions	GIT + feces	0.088 ± 0.041		0.119 ± 0.020	
^110m^Ag-ions	GIT + feces	0.873 ± 0.009		0.699 ± 0.062	
^192^Ir-ions	urine	0.277 ± 0.013	41.26	0.426 ± 0.112	6.911
^198^Au-ions	urine	0.087 ± 0.031	1.472	0.07 ± 0.01	0.350
^48^V-ions	urine	0.365 ± 0.077	4.127	0.319 ± 0.055	2.672
^110m^Ag-ions	urine	0.005 ± 0.006	0.006	0.009 ± 0.014	0.013

Comparison of fractional urinary and fecal excretions 24 h after IV injection and IT instillation of soluble ^192^Ir-ions [28], ^48^V-ions [30], ^198^Au-ions [25] and [^110m^Ag]Ag ions (cf. Figure 1) applied in the auxiliary studies in the present work. Note, in the data of 24 h fecal excretion, the 24 h GIT fractions are added due to the delayed passage through the GIT into feces. Data are given as mean ± SD, *n* ≥ 4 rats per group.

After IV injection of ^192^Ir-, ^198^Au- and ^48^V-ions these are minimally excreted in feces but more prominantly excreted via urine as indicated by their ratio (urinary: fecal) in Table 5. In contrast, IV injected [^110m^Ag]Ag ions are almost completely (fraction of0.87) excreted in feces and only a 100-fold lower amount in urine. This implies that [^110m^Ag]Ag^+^ ions do not remain in a form that allows renal clearance, which means that they either are no longer ions that could be excreted by renal clearance or that they have formed secondary nanoparticles which were not accessible to renal filtration. Instead, they were eliminated into the GIT for fecal excretion. This is in line with the findings in the literature as presented in the Background section and summarized above. Several reports [33, 39, 40, 55, 56] noted precipitation of Ag^+^-ions in biological fluids and subsequent formation of Ag-salt precipitates such as poorly soluble AgCl, Ag_2_S, Ag_2_PO_4,_ and Ag_2_Se. According to these observations and, since renal clearance of [^110m^Ag]Ag ions is negligible, after IV injection a large fraction of the Ag^+^-ions must have formed poorly soluble, nano-sized Ag-salt precipitates. The predominant fecally excreted fraction indicates that these precipitates were cleared via the hepato-biliary pathway; i.e. they were metabolized mainly by liver hepatocytes and released into the bile fluid of the Space of Dissé for further elimination via the gall bladder into the small intestine [57, 58] as sketched in Fig. 9. Note, hepatocytes do not metabolize metallic cations like [^110m^Ag]Ag [57, 58].

Twenty-four hours after IT instillation of ^192^Ir, ^198^Au and ^48^V ions only small fractions of between 0.06 and 0.2 (Table 5) are fecally excreted (including GIT retention), while 24 h after IT instillation of [^110m^Ag]Ag-ions, a fraction of 0.7 of the instilled dose is excreted in feces. This is almost the same fraction as after IV injection (0.87). Urinary excretion was similarly low after IV injection.

After IT instillation, rapid mucociliary clearance of [^110m^Ag]Ag deposited on the airway epithelium is expected to contribute to the fecally excreted fraction. However, based on the 24 h data after the inhalation of [^105^Ag]AgNP compiled in Table 3 it is not plausible to attribute a fraction of more than 0.3 to fast clearance up to this time point. According to literature presented in the Background section, it appears reasonable to attribute the additional fecal fraction of 0.3–0.4 to the rapid clearance of poorly soluble, nano-sized Ag-salt precipitates which had been formed with abundant Cl^−^-, S^2−^-, PO_4_^2−^-, ions as well as less abundant but more stably binding Se^2−^-ions present in ELF. For their rapid elimination via feces within 24 h, they were first translocated across the ABB into the blood and from there via the hepato-biliary pathway into the GIT and feces. Furthermore, after intratracheal instillation of [^110m^Ag]Ag -ions the differential fraction of 0.21 between the fecal excretion after 24 h (0.70, including the GIT content) and that after 7 days (0.91) is likely due to the hepato-biliary clearance pathway (HBC). Hence, there is a biphasic clearance after either IT instillation or IV injection of soluble [^110m^Ag]AgNO_3_. Additional confirmation comes from the daily fecal excretion measurements after all three instillation applications (IV, IT, GAV) which are shown in Fig. S7 of the Supplementary Information.

Since mucociliary clearance to the larynx and ABB translocation into blood lead both to fecal excretion of [^110m^Ag]Ag it is not directly possible to distinguish between both clearance pathways after IT instillation of soluble [^110m^Ag]Ag. The negligible amount of urinary excretion indicates that free ^110m^Ag-ions are virtually absent due to their precipitation in ELF, while a slightly higher fraction of ^198^Au-ions and large fractions of ^192^Ir- and ^48^V-ions were found in urine 24 h p.e. (see Table 5). Additionally, the third auxiliary biokinetics study in Fig. 1c after oral instillation of an [^110m^Ag]Ag-solution showed almost exclusive fecal and minimal urinary excretion, which is compatible with results reported earlier [40], and suggests the dominant precipitation of Ag-salt in the GIT and negligible uptake from the GIT through the intestinal barrier into the blood.

Therefore it is plausible to conclude that the differential 0.21 fraction is the upper limit of [^105^Ag]Ag-salt precipitates after [^105^Ag]AgNP inhalation which may have been translocated across the ABB from day 2 to day 7 into the blood and eliminated via HBC into the GIT and feces.

### Comparison of lung retention of inhaled, poorly soluble, 20 nm [^192^Ir]IrNP, [^195^Au]AuNP [^48^V]TiO_2_-NP and of inhaled, partially soluble, 20 nm [^105^Ag]AgNP

**Fig. 10:**
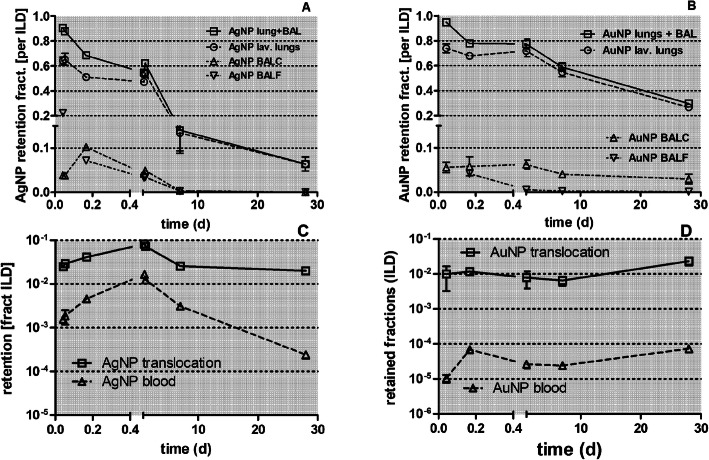
Comparison of lung retention of [^105^Ag]Ag activity after inhalation of [^105^Ag]AgNP with that of [^195^Au]Au activity after inhalation of [^195^Au]AuNP shown in panels** a** and **b**, respectively. Fractions are normalized to ILD. In panels** c **and **d** the total translocated [^105^Ag]Ag activity fractions across the ABB and the blood content after the inhalation of [^105^Ag]AgNP are compared with those after the inhalation of [^195^Au]AuNP. Mean (± SEM) for ^105^Ag]AgNP (*n* = 4) and *n* ≤ 6 for [^195^Au]AuNP

**Fig. 11:**
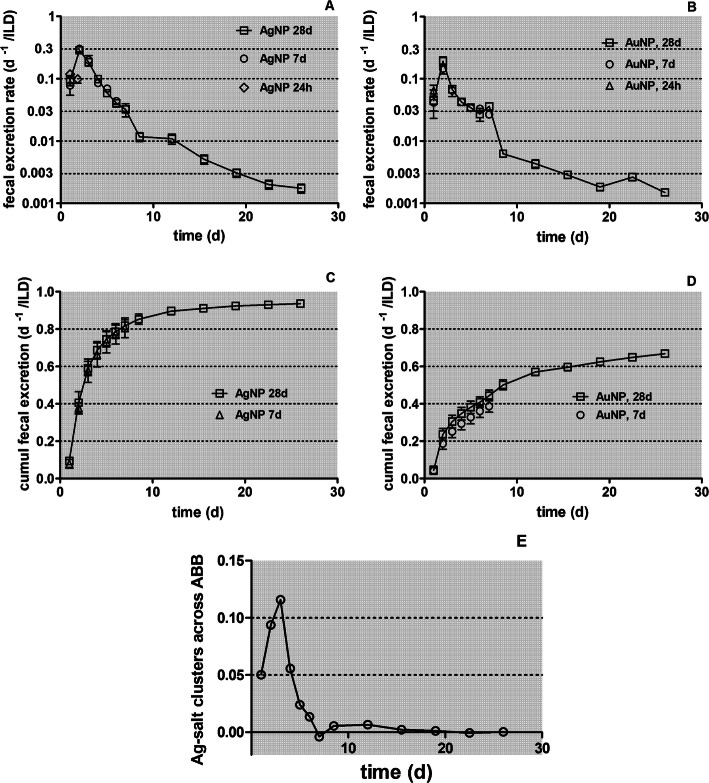
Fecal excretion of [^105^Ag]Ag and of [^195^Au]Au activity. Comparison of fecal excretion rates (panels** a** and **b**) and cumulative excretion (panels** c** and **d**), respectively. Fractional rates are normalized to ILD. For [^105^Ag]AgNP *n* = 4; for [^195^Au]AuNP *n* ≤ 6. In panel** e** the fecal excretion of [^105^Ag]Ag is shown which is attributed to precipitated [^105^Ag]Ag-salt, that had crossed the ABB into the blood and was eliminated via hepato-biliary clearance into the GIT. It is based on the difference between panels A and B assuming that both inhaled NP materials are cleared with similar fractional rates

**Fig. 12:**
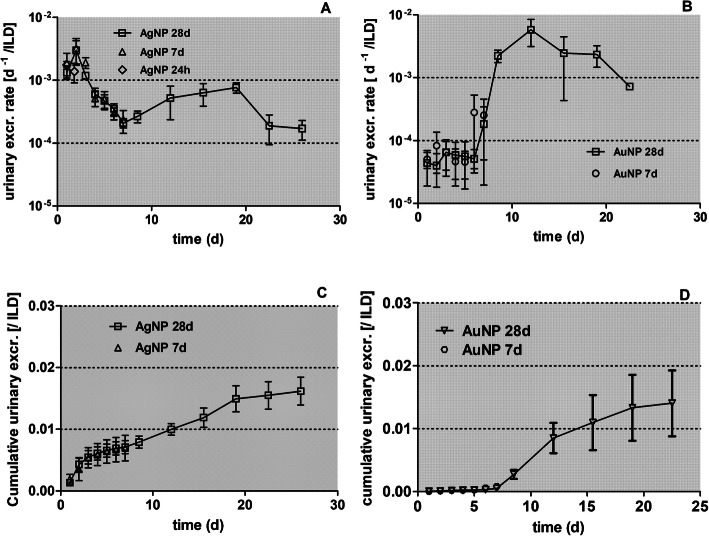
Comparison of urinary excretion after inhalation of [^105^Ag]AgNP and [^195^Au]AuNP. The urinary excretion rates and the cumulative urinary excretion of ^105^Ag (panel** a **and **c**, respectively) and ^195^Au (panel** b** and **d**, respectively) are shown. Data are normalized to ILD. For [^105^Ag]AgNP *n* = 4; for [^195^Au]AuNP n ≤ 6

**Fig. 13:**
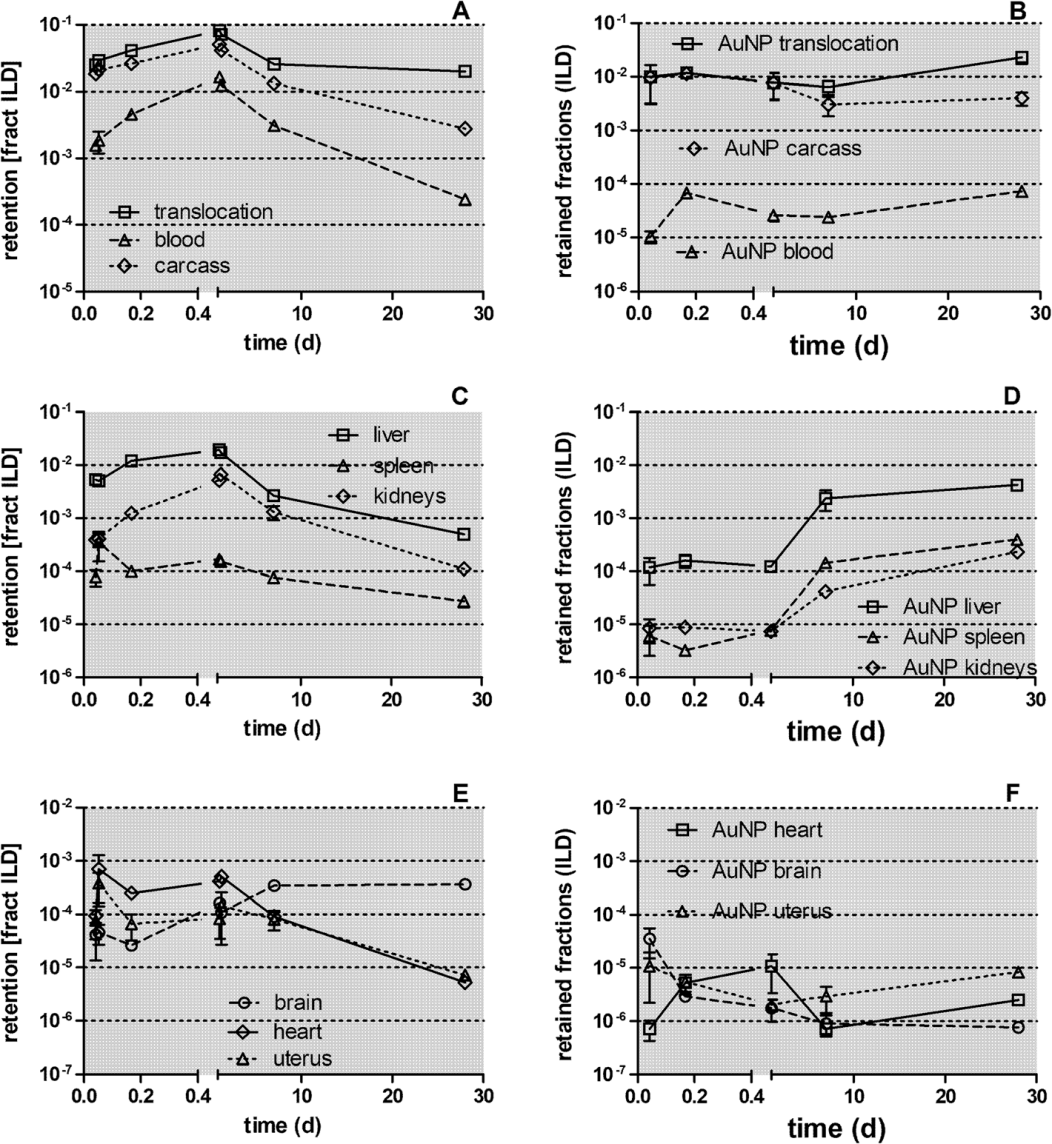
Comparison of retained [^105^Ag]Ag activity fractions in secondary organs and tissues after [^105^Ag]AgNP inhalation with those after [^195^Au]AuNP inhalation. Panels **a** and **b** show the activity fractions of total translocation, carcass, and blood; panels** c** and **d** show fractions for the liver, spleen, and kidneys; panels** e** and **f** show fractions for the heart, brain, and uterus. Fractions are normalized to ILD. Mean (± SEM) for [^105^Ag]AgNP inhalation (*n* = 4) and n ≤ 6 for [^195^Au]AuNP inhalation

**Fig. 14:**
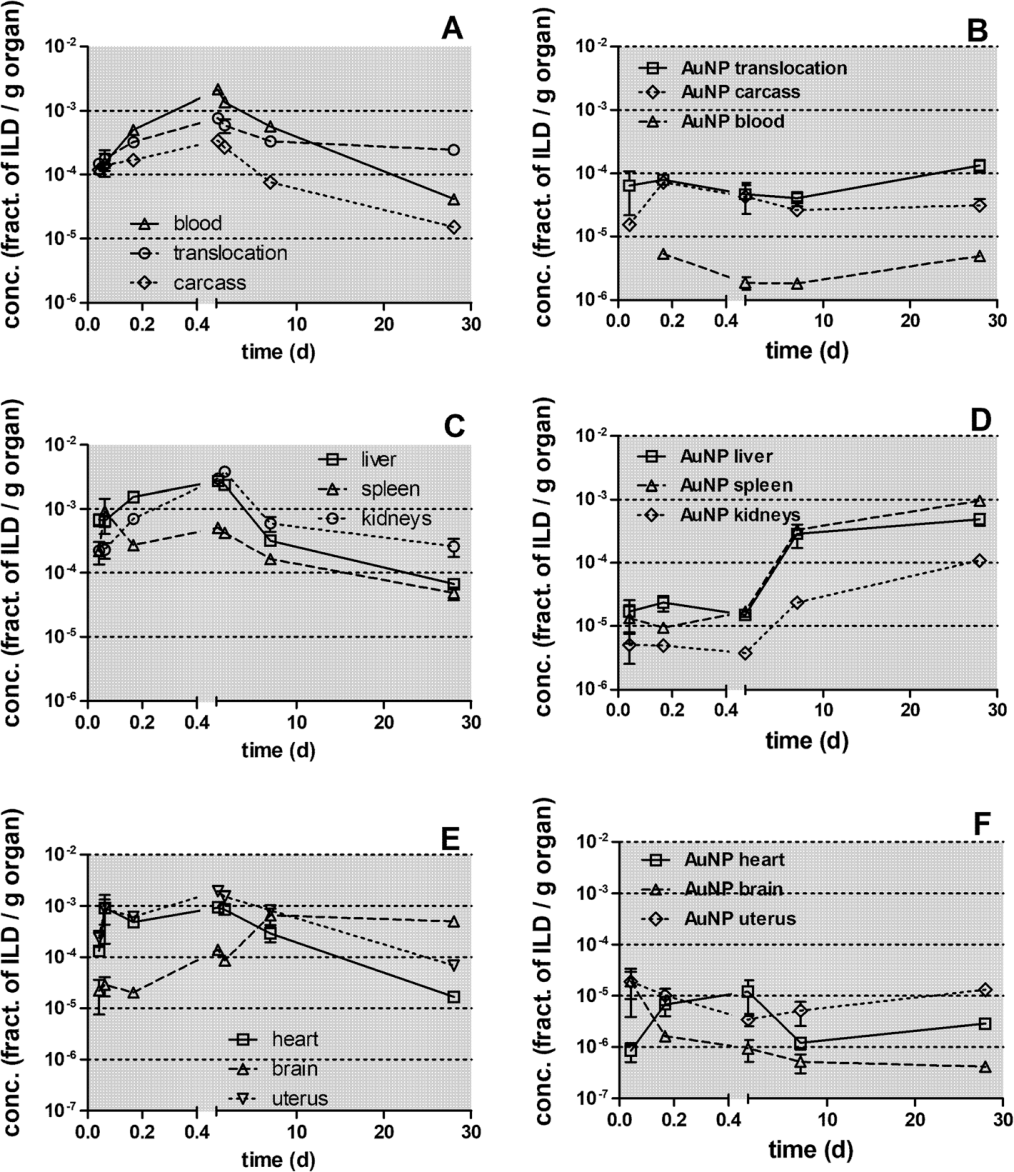
Comparison of retained fractional concentrations (/g organ) after [^105^Ag]AgNP inhalation with those after [^195^Au]AuNP inhalation in secondary organs and tissues. Panels **a** and **b** show fractional concentrations of total translocation, carcass, and blood; panels** c** and **d** show fractional concentrations of liver, spleen, and kidneys; panels** e** and **f** show fractional concentrations of heart, brain, and uterus. Fractional concentrations are normalized to ILD. Mean (± SEM) for [^105^Ag]AgNP inhalation (*n* = 4) and n ≤ 6 for [^195^Au]AuNP inhalation

Lung retention data of inhaled 20 nm [^105^Ag]AgNP differ strikingly from those previously obtained from poorly soluble 20 nm [^192^Ir]IrNP [28], 20 nm [^195^Au]AuNP [25] and 20 nm [^48^V]TiO_2_-NP [30]. While differences between the three inhaled, poorly soluble NP have been discussed previously, here we focus on the differences between [^105^Ag]AgNP and [^195^Au]AuNP in Figs. 10, 11, 12, 13 and 14 since both NP show a similar NP condensation dynamics immediately after spark ignition and evaporation. Initially, in the hot zone close to the igniting spark both, vaporized Ag and Au, condense and coalesce, forming liquid droplets up to 5–8 nm. Thereafter, when the droplets have escaped towards colder zones downstream, they solidify while continuing to coagulate until agglomeration is essentially stopped by dilution with clean air adjusted to maintain NP aerosol concentrations of about 1•10^7^ NP/cm^3^. The inhalation of these aerosols by the rats after sufficient cooling - occurs within 5–10 s after generation (see the experimental setup in Fig. S2 of Supplemental Information). Since we cannot distinguish later between the retention and clearance of the inhaled [^105^Ag]AgNP and their degradation products formed after inhalation we simply write “[^105^Ag]Ag activities”.

Figure 10b (adopted from [25]) shows that a fraction of 0.22 of the applied [^195^Au]AuNP dose is cleared from the lungs during the first 24 h after inhalation by MCC, while the cleared fraction of 0.4 for [^105^Ag]AgNP (see Fig. 10a) during the same period of time is nearly twice as high**.** We may reasonably assume that MCC will not only occur for the [^105^Ag]AgNP but also for their particulate degradation products – i.e. [^105^Ag]Ag-salt precipitates also formed on the airway epithelium. Assuming a similar mucociliary cleared fraction (of 0.22) of [^105^Ag]AgNP and precipitated [^105^Ag]Ag-salt as observed after [^195^Au]AuNP inhalation, the other half of the 0.4 decreases of [^105^Ag]Ag lung retention must be attributed to a second clearance pathway. Since the fraction of ^105^Ag retained in the blood after [^105^Ag]AgNP inhalation (Fig. 10c) during the first 24 h after inhalation is more than 100 times higher than the corresponding [^195^Au]Au activity after [^195^Au]AuNP inhalation (Fig. 10d), we conclude that translocation of [^105^Ag]AgNP and their degradation products across the ABB accounts for this cleared fraction. From the blood, the material is eliminated via hepato-biliary clearance into the GIT and subsequently excreted in feces similar to the fecal clearance after IT instillation of soluble [^110m^Ag]AgNO_3_.

Similarly, Fig. 10a and b show that from day 2 until day 7, long-term macrophage-mediated clearance (LT-MC) of [^195^Au]AuNP causes a fractional decrease of lung retention by 0.2, while after inhalation of [^105^Ag]AgNP the fractional lung retention decreases by 0.45. This suggests an association of a 0.25 fraction to the translocation pathway of the precipitated [^105^Ag]Ag-salt across the ABB which is supported by the persisting 100-fold higher [^105^Ag]Ag content in blood when compared to the circulating [^195^Au]AuNP.

### Comparison of the fecal excretion after inhalation of [^105^Ag]AgNP and [^195^Au]AuNP

The daily fecal excretion per ILD and the cumulative fecal excretion after inhalation of [^105^Ag]AgNP are shown in Fig. 3a and b. Following the arguments above, the [^105^Ag]Ag^+^-ions precipitate rapidly in the ion-rich ELF forming nano-sized silver salt precipitates of [^105^Ag]AgCl_3_, [^105^Ag]Ag_2_S and/or [^105^Ag]Ag_2_Se. In addition to [^105^Ag]AgNP deposited in the airways, also silver salt precipitates formed in the conducting airways are expected to undergo mucociliary clearance towards the larynx to be swallowed into the GIT. After [^105^Ag]AgNP deposition in the lungs, ionic [^105^Ag]Ag -release continues over extended time periods since salt layers formed on the [^105^Ag]AgNP surface slow down [^105^Ag]AgNP dissolution [31, 34, 35, 38].

In Fig. 11 daily and cumulative fecal excretion data for [^105^Ag]Ag and [^195^Au]Au after inhalation of [^105^Ag]AgNP and [^195^Au]AuNP, respectively, are compared for the groups of rats dissected at retention time points 24 h, 7 days and 28 days p.e. All data are normalized to the ILD. The dynamics of excretion rates in panels A and B look rather similar, although the maximum excretion rates of [^105^Ag]Ag and of [^195^Au]Au 2 days p.e. are 0.3 and 0.2 d^− 1^ relative to ILD, respectively. This difference becomes apparent in panels C and D which show the cumulative excretion data. After 28 days the cumulative excreted fraction of [^105^Ag]Ag is 0.94 of ILD, while that of [^195^Au]Au is 0.66 of ILD; i.e. only two-thirds of [^195^Au]Au are excreted when compared to [^105^Ag]Ag. Already within 2 days after inhalation, the cumulated excretion of [^105^Ag]Ag (0.4 of ILD) is twice as high as the corresponding value for [^195^Au]Au (0.2 of ILD) and the difference of a factor of two persists 7 days after inhalation with values of 0.8 and 0.4, respectively. Similar to the findings after intratracheal instillation of soluble [^110m^Ag]Ag-ions (see Fig. 1), we postulate that besides mucociliary clearance from the conducting airways a second clearance pathway was possible after inhalation of [^105^Ag]AgNP via ABB translocation into the blood and subsequent clearance into the duodenum via HBC. The contribution of this additional clearance pathway can be estimated by assuming a similar clearance for [^105^Ag]AgNP and [^195^Au]AuNP in Fig. 11c and d. The derived calculation is provided by Eqns. 21 and 22 of the Supplementary Information. The difference between these two cumulative excretion curves is shown in Fig. 11E and can be attributed to slowly dissolving [^105^Ag]Ag-salt precipitates cleared via ABB translocation. It shows a steep increase up to a fraction of 0.12 reached at 3 days p.e. after which the [^105^Ag]Ag-salt translocation diminishes rapidly reaching about 0.007 at 12 days p.e. Thereafter no further [^105^Ag]Ag-salt translocation occurs and fecal excretion rates of [^195^Au]AuNP and of [^105^Ag]AgNP are similar. This indicates that after 2 weeks p.e. there are still undissolved persistent cores of [^105^Ag]AgNP most likely protected by slowly dissolving Ag-salt layers as observed in previous studies [31, 40, 41] and discussed in the Background section of this report.

### Comparison of the urinary excretion after inhalation of [^105^Ag]AgNP and [^195^Au]AuNP

As shown in Fig. 4, [^105^Ag]Ag activity fractions in urinary excretion are very low and accumulate after 28 days to a fraction of only 0.015 of the inhaled [^105^Ag]AgNP dose. When comparing these data with those after inhalation of poorly soluble [^195^Au]AuNP in Fig. 12 there are clear differences in the kinetics of urinary excretion. After inhalation of [^105^Ag]AgNP, a maximal urinary excretion rate of 0.003 d^− 1^ is observed 2 days p.e. followed by a decrease of a factor of 10 until the end of the observation period 28 days p.e. We hypothesize that the urinary excretion pattern during the first week p.e. relates to the urinary excretion of very small amounts of nano-sized precipitated [^105^Ag]Ag-salt which are smaller than 6–8 nm and will pass through the kidneys into urine [52, 59].

In contrast, after [^195^Au]AuNP inhalation the urinary excretion rates remain very low at about 0.00005 d^− 1^ throughout the first week p.e. and start to increase thereafter with a peak fraction of 0.005 at 12 days p.e. As a result, the cumulative urinary excretion of inhaled [^195^Au]AuNP in Fig. 12d is almost negligible during the first week p.e. but increases steadily up to 0.015. In contrast, cumulative urinary excretion of inhaled [^105^Ag]AgNP in Fig. 12c steadily increases after inhalation up to 0.017 at 28 days p.e., which is similar to that after [^195^Au]AuNP inhalation in Fig. 12d.

### Comparison of retained fractions in secondary organs and tissues after inhalation of [^105^Ag]AgNP and [^195^Au]AuNP

In Fig. 13 the fractional retention of [^105^Ag]Ag activities in secondary organs and tissues (taken from Fig. 5) is compared with the corresponding data after inhalation of [^195^Au]AuNP. For the latter, these fractions result from the translocation of [^195^Au]AuNP across the ABB into the blood while after inhalation of [^105^Ag]AgNP the nanoparticles as well as [^105^Ag]Ag-salt precipitates, forming as degradation products of the [^105^Ag]AgNP when releasing Ag^+^ ions, will contribute to ABB translocation. Strikingly, the [^195^Au]AuNP fractions in the blood (see Fig. 13b) remain below 0.0001 throughout the study period, while after inhalation of [^105^Ag]AgNP in Fig. 13a the [^105^Ag]Ag activity fraction retained in blood is more than tenfold higher and reaches a maximum of 0.01 within 24 h p.e. This huge difference indicates a major contribution of precipitated [^105^Ag]Ag-salt in the blood, which appears to decrease rapidly after 24 h and approaches similarly low levels as those observed 28 days after [^195^Au]AuNP inhalation.

This is consistent with the diminished fractions of precipitated, fecally excreted [^105^Ag]Ag attributed to [^105^Ag]Ag-salt in Fig. 11e. The total translocated fractions are several-times higher after inhalation of [^105^Ag]AgNP than those observed after [^195^Au]AuNP inhalation (see Fig. 13a and b). If we assume that the [^195^Au]AuNP and the persisting, but probably smaller (by dissolution) [^105^Ag]AgNP behave in the same way, the almost 100-fold higher [^105^Ag]Ag activity fractions in liver and kidneys point to a rapid removal of precipitated [^105^Ag]Ag-salt from the blood by these two organs (see Figs. 13c and d). The rapid clearance of [^105^Ag]Ag activity from the blood and the increased values of [^105^Ag]Ag in the spleen, heart, brain, and uterus during the first 24 h p.e. shown in Fig. 13e should then also be attributed to uptake of [^105^Ag]Ag-salt precipitates.

### Comparison of retained fractions per mass of secondary organs and tissues after inhalation of [^105^Ag]AgNP and [^195^Au]AuNP

When comparing the fractional concentrations per weight of each secondary organ or tissue after inhalation of [^105^Ag]AgNP and [^195^Au]AuNP in Fig. 14, the initial fractional concentrations after[^105^Ag]AgNP inhalation are up to two orders of magnitude higher than after inhalation of [^195^Au]AuNP. Assuming that the cores of the [^105^Ag]AgNP and [^195^Au]AuNP are translocated and distributed similarly, the observed differences should be attributable to the translocation and the retention of precipitated [^105^Ag]Ag-salt. Of note,during the first 24 h p.e. the fractional concentrations of all secondary organs (except the brain for [^105^Ag]AgNP), remain in a narrow range of one order of magnitude for each type of nanoparticle, respectively, (compare Fig. 14c and d and Fig. 14e and f). This may indicate rather a rather slow clearance dynamics in the secondary organs. Along this line the similar fractional concentrations of both types of nanoparticles in the brain suggests that only a small amount of precipitated [^105^Ag]Ag-salt accumulates in the brain during the first 4 h p.e. But 24 h and 7 days p.e. the [^105^Ag]Ag accumulation in the brain increases rather rapidly which differs from the rather constant fractional concentrations of the other secondary organs. This may suggest that the blood-brain-barrier is tight for [^105^Ag]Ag-salt precipitates only for the first 4 h p.e.

## Conclusion

Intratracheal inhalation of freshly generated [^105^Ag]AgNP aerosols for 1½ hours allows low dose deposition in the lungs of adult healthy rats thereby avoiding nasal and pharyngeal deposition. The sensitivity obtained with radiolabeled NP is extremely high. Highly sensitive γ-ray-spectrometry, allows a dynamic, analytical range over five orders of magnitude across the different specimens. The methodology of radio-analysis is easy to manage for the large number of samples in order to study quantitative biodistributions of [^105^Ag]AgNP and their possible degradation products in the rat organism, as well as complete excretional clearance out of the rat organism after five different retention time intervals up to 28 days p.e. The repeatability of the initially rapidly evolving biokinetics proved to be convincingly good when comparing the two independently investigated groups of four rats studied at 0.75 h and 24 h p.e., respectively.

Rapid release of [^105^Ag]Ag-ions from the [^105^Ag]AgNP surface appears to be superimposed with both fast MCC from the conducting airways and long-term clearance of [^105^Ag]AgNP from the alveolar region to the larynx. The current observations are in line with evidence presented in the literature that released [^105^Ag]Ag-ions precipitate rapidly to form very small clusters of low solubility [^105^Ag]Ag-salts in the ion-rich ELF. Besides their clearance to the larynx, they translocate across the ABB into the blood and are predominantly eliminated via the hepato-biliary clearance pathway into the small intestine for fecal excretion. The pathway of precipitated [^105^Ag]Ag-salt is supported by the findings of auxiliary biokinetics studies at 24 h and 7 days after either IV injection or IT or oral instillation of [^110m^Ag]AgNO_3_ solutions in sentinel groups of rats. In line with evidence in the literature, the release of [^105^Ag]Ag-ions from the [^105^Ag]AgNP surface may continue for an extended time at a lower rate due to the simultaneous formation of low-solubility salt layers on the [^105^Ag]AgNP surface in the ion-rich body fluids that mediate and prolonge the dissolution process.

The superimposed clearance of (possibly) Ag-salt-layer-protected, persistent cores of [^105^Ag]AgNP and precipitated [^105^Ag]Ag-salt results in the elimination of a fraction of > 0.8 (per ILD) after one-week p.e. Assuming a very similar clearance behavior of the cores of AgNP and AuNP the difference between the results of both experiments can be attributed to the clearance of Ag-precipitates during the first 2 weeks p.e., showing a maximal fractional rate of 0.12 d^− 1^ at 3 days p.e.

## Materials and methods

### Study design

Twenty-eight healthy, adult, female Wistar Kyoto rats (WKY/Kyo@Rj rats, Janvier, Le Genest Saint Isle, France) were randomly assigned to seven groups of four rats and subjected to intratracheal inhalation of an [^105^Ag]AgNP aerosol for 1½ hours via an endotracheal tube [60]. The biodistribution was analyzed 0.75 h, 4 h, 24 h, 7d and 28d after exposure. The first group of rats, which was exsanguinated and dissected immediately after the 1½-hour intratracheal inhalation exposure, was assigned to the retention time-point 0.75 h since the nanoparticles brought into the rats’ lungs over 1½ hours had an estimated average time of only 45 min for deposition, uptake, and distribution. In order to test the repeatability of the rapidly changing biokinetics directly after inhalation, two additional groups of four rats were inhalation exposed and analyzed at 0.75 h and 24 h p.e., respectively. The additional two groups were exposed 1 day after the inhalation exposures of the initial 0.75 h and 24 h groups, respectively.

Furthermore, twenty-four healthy, adult, female Wistar Kyoto rats (WKY/Kyo@Rj rats, Janvier, Le Genest Saint Isle, France) were randomly assigned to six groups of four rats and subjected to intratracheal instillation, or intravenous injection, or oral instillation (gavage) of soluble [^110m^Ag]AgNO_3_ solutions and biokinetically studied at either 24 h or 7 days p.e.

### Animals and maintenance

All Wistar-Kyoto rats (WKY/Kyo@Rj rats, Janvier, Le Genest Saint Isle, France) were housed in relative-humidity and temperature-controlled ventilated cages (VentiRack Bioscreen TM, Biozone, Margate, UK) on a 12-h day/night cycle. Rodent diet and water were provided ad libitum. The rats were adapted for at least 2 weeks after purchase and then randomly attributed to the experimental groups. When starting the studies, the rats were 8–10 weeks old and exhibited a mean body weight of 204 ± 13 g. Some physiological parameters of the rats are given in Table 3.

All experiments were conducted under German federal guidelines for the use and care of laboratory animals in accordance with EU Directive 2010/63/EU for animal experiments. The studies were approved by the Regierung von Oberbayern (Government of District of Upper Bavaria, Approval No. 211–2531-94/04) and by the Institutional Animal Care and Use Committee of the Helmholtz Centre Munich.

### Synthesis and characterization of the [^105^Ag]AgNP aerosol

Radiolabeled AgNP aerosols were produced continuously during the experiments by spark ignition between two high-purity (99.997%), cylindrical silver electrodes (diameter 3.0 mm, length 5 mm; Cat. No. AG007912; Goodfellow GmbH, Hamburg, Germany) which had been proton irradiated in the cyclotron at JRC (Ispra, Italy). The protons impinging on one of the flat ends of each electrode had an energy of about 33.5 MeV in order to achieve the highest possible ^105^Ag-activity near the surface. This radioactivity was distributed nearly homogeneously throughout a layer thickness that would theoretically be consumed by the spark ignition process during the experiments. At the time the inhalation experiments were performed, the specific ^105^Ag radioactivity was 2.60 MBq/mg in a surface layer of about 250 μm thickness. The radioactive ^105^Ag decays back to ^105^Pd via electron capture and positron emission, with a half-life of 41.3 days, thereby emitting γ-rays with different energies; one γ-emission line of 0.28 MeV with a fractional emission probability of 0.302 was selected for the γ-spectrometrical analyses.

For each group of rats, the [^105^Ag]AgNP aerosol was freshly generated in the spark ignition aerosol generator (GFG100, Palas, Karlsruhe, Germany) at 250 Hz spark frequency in an argon (Ar) gas stream of 3 L/min. During spark ignition between the two Ag electrodes, small amounts of Ag evaporate. The vaporized Ag coalesces, forming liquid droplets up to 5–8 nm. When the droplets have escaped towards colder zones downstream, they solidify to solid spheres. The electrically charged aerosol of these primary spheres is immediately quasi-neutralized by an inline radioactive ^85^Kr source and the highly concentrated and continuously agglomerating [^105^Ag]AgNP pass through a 30 cm long tubular furnace that is kept at a temperature of 600 °C to form single, densely packed agglomerated particles. Coagulation continues until it is stopped by dilution with clean air when the [^105^Ag]AgNP reach a size of 20 nm. Since densely packed spheres will occupy 74% and more randomly packed spheres about 64% of the agglomerate volume we suggest a density of 7.3 g/cm^3^ (= 0.7 • 10.49) of the generated and heat-treated AgNP.

Downstream of the furnace the aerosol was cooled and diluted in a copper tube (inner diameter 8 mm) by mixing with humidified oxygen and nitrogen to obtain a fractional oxygen concentration of 0.2–0.25. After dilution to concentrations of about 1•10^7^ NP/cm^3^ further agglomeration was negligible within the few seconds prior to inhalation by the rats. The generated 20 nm-sized [^105^Ag]AgNP still have a chain agglomerated/aggregated structure even after 600 °C heat-treatment albeit more compact than the non-heat-treated NP. The flow rate was typically 10 L/min and the fractional relative humidity of the aerosol was set to about 0.7 before entering the inhalation apparatus; the whole inhalation apparatus and the inhalation methodology including the pre-set fractional relative humidity were described earlier [61] and is schematically displayed in Fig. S3 of the Supplementary Information. The aerosol particle concentration and size distribution were continuously sampled and controlled by a condensation particle counter (CPC 3022A, TSI, Aachen, Germany) and a scanning mobility particle spectrometer (SMPS; consisting of a model 3071 differential mobility analyzer and a CPC model 3010, TSI, Aachen, Germany), respectively. Averages of the count median diameters (CMD), volume median diameters (VMD) and geometric standard deviations (GSD) as well as number concentrations and volume concentrations are given as mean ± SD in Table 2. Since the SMPS instrument exhibited a lower particle size detection limit of 10 nm, the averaged spectra were fitted to a lognormal size distribution using the least-squares method and the fits were extrapolated to a size of 1 nm (for details see Supplementary Information). These corrections led to slightly lower CMDs while the GSD changed only negligibly (see Table 2). The characteristic parameters of the freshly generated [^105^Ag]AgNP aerosol were the same as those generated without the radio-label using non-irradiated, pure silver electrodes; TEM images were analyzed from the latter. Similarly, the chemical composition was determined by Electron Energy Loss Spectrometry (EELS). The specific ^105^Ag activity of the aerosol particles was determined by γ-spectrometric analysis of absolute filters onto which [^105^Ag]AgNP had been collected at an aerosol flow (0.3 L/min) throughout each 1½-h exposure period. From the activity deposited on the filter, an activity concentration of the [^105^Ag]AgNP aerosol of 2.60 kBq•μg^− 1^ was derived. At this activity concentration the atomic ratio of ^105^Ag: Ag in the nanoparticles is about 1.3 × 10^− 6^. Hence, statistically, every second AgNP will contain a ^105^Ag -radiolabel. Therefore, the ^105^Ag-radiolabeling involves a minimal impurity of the stable silver matrix which would be extremely unlikely to affect the stability and the physicochemical characteristics of the [^105^Ag]AgNP.

### Intratracheal inhalation exposure

The four slightly anesthetized adult rats in each group were ventilated individually via a flexible endotracheal tube and placed on their left lateral side in an air-tight plethysmograph box of our tailor-made inhalation apparatus and connected to the aerosol system, (see Fig. S3 of Supplementary Information). They were exposed to the freshly generated aerosol for 1½ hours. In this report, this exposure method will be called “intratracheal inhalation” (see reference [60]).

### Treatment of the rats after inhalation

Anesthesia of each rat was antagonized immediately post-exposure (p.e.) as described in the Supplementary Information and previously in [25, 62]. Thereafter, each rat was kept individually in a metabolic cage and excreta were collected separately and quantitatively. For ethical reasons, the rats of the 28 days group were maintained individually in a normal cage on cotton cloths starting immediately after [^105^Ag]AgNP inhalation. Each cloth was replaced with a new one every 3–4 days (2 cloths per week), and the fecal droppings were quantitatively separated from the previous one. After separation, the cloth contained only [^105^Ag]Ag activity originating from urine which had soaked and dried. In Table S2 the list of collected organs, tissues, body fluids, and excretion is given. Since the cages of the four rats of each group were located next to each other, the rats had the continuous sensory perception of each other.

### Evaluation and statistical analysis of [^105^Ag]AgNP biokinetics

At 0.75 h, 4 h, 24 h, 7d, and 28d p.e., rats were anesthetized (by 5% isoflurane inhalation) and euthanized by exsanguination via the abdominal aorta. Blood, all organs, tissues, and excretions were collected and the ^105^Ag radioactivity was determined by γ-spectrometry without any further physicochemical processing of the samples, as described in the Supplementary Information and in earlier works [25, 62–64]. Throughout this report nanoparticle quantities are calculated from the ^105^Ag activity determined with γ-scintillation detectors, properly calibrated in γ-ray energy and detection efficiency for ^105^Ag, and corrected for background and radioactive decay during the experiments (see Supplementary Information). Samples yielding background-corrected counts in the 280 keV region-of-interest of the ^105^Ag γ-spectrum were defined to be below the detection limit (<DL; 0.1 Bq) when the number of counts was less than three standard deviations of the background counts collected during 200 min without any sample in the γ-scintillation detector.

BALs were performed by applying 6 × 5 ml of phosphate-buffered-saline solution (PBS without Ca^2+^ or Mg^2+^) under the gentle massage of the thorax. The recovered fractional BAL fluid (BALF) (about 0.8 of instilled PBS) was centrifuged at 500 g for 20 min at room temperature to separate the lavaged cells from the supernatant. The [^105^Ag]AgNP content was determined by γ-ray-spectrometry.

Up to 24 h p.e. early clearance was measured in the GIT and feces comprising of (a) MCC-cleared [^105^Ag]AgNP and of precipitated, low-solubility [^105^Ag]Ag-salt precipitates from the conducting airways and (b) of precipitated, low-solubility [^105^Ag]Ag-salt precipitates which had translocated across the respiratory ABB into blood and via liver and gall-bladder into the small intestine. The early clearance data of the 7 days- and 28 days-group were derived from fecal excretion measurements during the first 3 days p.e. The clearance contribution of the [^105^Ag]Ag^+^-ion release - according to the literature discussed in the Background section - precipitating as low-solubility salt precipitates was calculated from the 24 h data of the auxiliary study after the intratracheal instillation of [^110m^Ag]Ag solution.

After 3 days p.e. fecal excretion was considered as long-term clearance from the peripheral lungs comprising of macrophage-mediated LT-MC of (a) persistent, low-solubility Ag-salt-layer protected cores of [^105^Ag]AgNP in the lung periphery towards the larynx into the GIT and (b) low-solubility [^105^Ag]Ag-salt precipitates translocated across the ABB into blood and via liver and gall-bladder into the small intestine. The clearance contribution of the long-term [^105^Ag]Ag^+^-ion release precipitating to low-solubility salt precipitates was estimated from the difference of the 7 days minus 24 h data of the auxiliary study after the intratracheal instillation of [^110m^Ag]Ag solution.

About 0.7 of the fractional blood volume was recovered by exsanguination. Thus, organs and tissues contain residual blood whose ^105^Ag radioactivity needs to be subtracted to obtain the true content of nanoparticles and their degradation products. For this purpose, the residual blood contents of organs and tissues after exsanguination were calculated by making use of the findings of Oeff & König [48], and the true radioactivities of the organs and tissues were obtained by subtracting the blood-related ^105^Ag-radioactivity values. The procedure is outlined (see Eqns. (4–8)) in the Supplementary Information.

The measured [^105^Ag]Ag-activity values were expressed as fractions of the initial lung dose (ILD) i.e. the [^105^Ag]AgNP radioactivity deposited in the lungs. Fractions were normalized to the sum of all sampled ^105^Ag-radioactivities of a given rat (see Supplementary Information). The mathematical procedure is derived in Eqns. 11 and 14 of the Supplementary Information. The fractions for each organ or tissue were averaged over the group of rats and were presented with the standard error of the mean (SEM). All calculated significances are based on One-Way-ANOVA analyses with the post-hoc Bonferroni test. In the case of direct comparisons of two groups, the unpaired t-test was used. Significance was considered at *p* ≤ 0.05.

The biokinetics data of lung-applied [^105^Ag]AgNP were further normalized to the [^105^Ag]AgNP fraction which had translocated across the ABB (see Eqns. 23 and 24 in the Supplementary Information).

### Biokinetics of soluble [^110m^Ag]Ag after intratracheal instillation, intravenous injection or oral instillation (gavage)

Since [^105^Ag]AgNP reportedly dissolve in body fluids a certain fraction of the [^105^Ag]Ag may be released from the [^105^Ag]AgNP, thus affecting the AgNP biokinetics analysis. In order to estimate such a release from the [^105^Ag]AgNP surface and to quantify its effect, auxiliary experiments with carrier-free ionic ^110m^Ag were carried out. Note, that we could not use ^105^Ag for these studies since this radio-isotope was produced during proton bombardment of the metallic matrix of the solid silver electrodes using the JRC cyclotron; instead, we used ^110m^Ag^+^ in a 0.1 m HNO_3_ solution (5 mg Ag^+^ in 0.5 ml) which was neutron-activated at the nuclear research reactor of Research Centre Rez (Husinec-Rez, Czech Republic) yielding 24 MBq of ^110m^Ag^+^. The ^110m^Ag isotope has a half-life of 249.9 d and is a gamma emitter emitting with several gamma emission lines of which we used those in the range of 650–900 keV. The use of both ^110m^Ag and ^105^Ag isotopes of the chemical element Ag is expected to be equivalent in these biokinetics studies since both are isotopes of the chemical element silver. As reported previously [62], auxiliary studies (AUX) were performed at 24 h and 7 days after intratracheal instillation (IT) or intravenous injection (IV) or oral instillation (gavage) with the purpose of correcting the biodistributions of inhaled [^105^Ag]AgNP for [^105^Ag]Ag -ion release. In order to mimic [^105^Ag]Ag released by [^105^Ag]AgNP in the auxiliary studies, the following [^110m^Ag]Ag^+^-solutions were prepared: 0.33 μg•μL^− 1^ ionic Ag(NO_3_) was added to carrier-free ionic ^110m^Ag^+^. The pH value was adjusted to 5. For the experiments, 60 μL of a solution containing 100 kBq ionic ^110m^Ag^+^ and 20 μg of ionic, non-radioactive Ag^+^ were administered per rat.

## Supplementary information

**Caption of Additional File 1 .**

## Data Availability

The raw/processed data required to reproduce these findings cannot be shared at this time due to technical or time limitations. The datasets supporting the conclusions of this article are included within the article and its additional file. Details of the supplementary information associated with this paper are provided at the end of this manuscript document under “Additional File 1”.

## Abbreviations

*: Level of significance *p* < 0.05;
**: Level of significance *p* < 0.01;
***: Level of significance *p* < =0.001;
ABB: AIR-blood barrier
AgNP, [^105^Ag]AgNP: Silver nanoparticles without or with ^105^Ag radiolabel
AgNO_3_, [^110^Ag]AgNO_3_: Silver nitrate solution without or with ^110^Ag radiolabel
AuNP, [^195^Au]AuNP: Gold nanoparticles without or with ^195^Au radiolabel
BAL: Broncho-alveoar-lavage
BALC: Cells of BAL
BALF: Fluid of BAL
CPC: Condensation particle counter
ENV: Exosomal nanovesicle
GAV: Esophageal instillation (gavage)
GIT: Gastro-intestinal-tract
HBC: Hepato-biliary clearance
ILD: Initial lung dose
IPLD: Initial peripheral lung dose
IrNP, [^192^Ir]IrNP: Iridium nanoparticle without or with ^192^Ir radiolabel
IT: Intratracheal instillation
IV: Intravenous injection
Lav. Lungs: Lavaged lungs
LTMC: Long-term macrophage-mediated particle clearance
Lung-BAL: Total lungs being the sum of the lav. Lungs and BALC and BALF and Tra-bron
ns: Not significant
p.e.: Post-exposure
MCC: Mucociliary clearance
SMPS: Scanning mobility particle sizer
TEM: TRANSMISSION electron microscope
TiO_2_NP, [^48^V]TiO_2_NP: Titanium dioxide without or with ^48^V radiolabel
Tra-bron: Sample of the trachea and the two main bronchi
