# Supplementary material for: Quantitative biokinetics over a 28 day period of freshly generated, pristine, 20 nm silver nanoparticle aerosols in healthy adult rats after a single 1½-hour inhalation exposure
Source: Part Fibre Toxicol. 2020 Jun 5;17:21. doi: 10.1186/s12989-020-00347-1 (PMC7275317; doi:10.1186/s12989-020-00347-1)
Supplement: Supplementary file 1 — Caption of Additional File 1 . [file 12989_2020_347_MOESM1_ESM.docx]

## Supplementary Information

**Quantitative biokinetics over a 28 day period of freshly generated, pristine, 20 nm silver nanoparticle aerosols in healthy, adult rats after a single 1½-hour inhalation exposure**

Wolfgang G. Kreyling*^1,2^ Uwe Holzwarth^3^, Stephanie Hirn^1^, Carsten Schleh^1,4^, Alexander Wenk^1,5^, Martin Schäffler^1^, Nadine Haberl^1^, Neil Gibson^3^

^1^ Comprehensive Pneumology Center, Institute of Lung Biology and Disease, Helmholtz Zentrum München – German Research Center for Environmental Health, Ingolstaedter Landstrasse 1, D-85764 Neuherberg / Munich, Germany

^2^ Institute of Epidemiology, Helmholtz Center Munich – German Research Center for Environmental Health, Ingolstaedter Landstrasse 1, D-85764 Neuherberg / Munich, Germany

^3^ European Commission, Joint Research Centre (JRC), Ispra, Italy

^4^ Current address: Abteilung Gesundheitsschutz, Berufsgenossenschaft Holz und Metall, Am Knie 8, D-81241 München, Germany

^5^ Current address: Dept. Infrastructure, Safety, Occupational Protection, German Research Center for Environmental Health, Ingolstaedter Landstrasse 1, D-85764 Neuherberg / Munich, Germany

***Corresponding author**Dr. Wolfgang G. Kreyling
Institute of Epidemiology
Helmholtz Centre Munich, German Research Center for Environmental Health
Ingolstaedter Landstrasse 1,

85764 Neuherberg / Munich, Germany
Email: [kreyling@helmholtz-muenchen.de](mailto:kreyling@helmholtz-muenchen.de),

Phone: +49-89-2351-4817

**Email addresses of the authors:**

[kreyling@helmholtz-muenchen.de](mailto:kreyling@helmholtz-muenchen.de)

[Uwe.HOLZWARTH@ec.europa.eu](mailto:Uwe.HOLZWARTH@ec.europa.eu)

[Carsten.Schleh@bghm.de](mailto:Carsten.Schleh@bghm.de)

[stephanie.hirn@helmholtz-muenchen.de](mailto:stephanie.hirn@helmholtz-muenchen.de)

[alexander.wenk@helmholtz-muenchen.de](mailto:alexander.wenk@helmholtz-muenchen.de)

[MSBiology@gmx.de](mailto:MSBiology@gmx.de)

[haberl.nadine10@googlemail.com](mailto:haberl.nadine10@googlemail.com)

[Neil.GIBSON@ec.europa.eu](mailto:Neil.GIBSON@ec.europa.eu); [neilgibson15@gmail.com](mailto:neilgibson15@gmail.com)

The supplementary information is available free of charge on the BMC website.

**Content**

1. Aerosol size distribution measurements using the SMPS and spectral fitting

## In vitro dissolution of [^105^Ag]AgNP submersed in distilled water

1. Intratracheal inhalation exposure to the freshly generated [^105^Ag]AgNP aerosol
2. Radiometric and statistical analysis
3. Parameters of inhalation and deposition
4. Preparation of biokinetics samples for radiometric analysis

## Blood correction and total blood volume

1. Total [^105^Ag]AgNP deposition in each rat determined by the balanced [^105^Ag]Ag activities of the entire dissected rat including its total excretion
2. Lung and body retention fits
3. Bronchoalveolar lavage (BAL)

## Estimated clearance rates of translocated [^105^Ag]Ag-salt clusters across the ABB into the blood circulation and GIT

## [^105^Ag]AgNP accumulation and retention in secondary organs and tissues relative to translocated [^105^Ag]AgNP across the ABB

## Auxiliary biokinetics studies after application of soluble [^110m^Ag]Ag(NO_3_)_3_ applications

1. References

## Aerosol size distribution measurements using the SMPS and spectral fitting

For each 1.5-hour inhalation exposure, the [^105^Ag]AgNP size distribution was measured continuously by SMPS resulting in about 30 spectra. The SMPS (consisting of a model 3071 Differential Mobility Analyzer and a model 3010 CPC, TSI, Aachen, Germany) was operated at a main flow of 6 L/min and a sample flow of 0.6 L/min providing spectra in the range of 10 nm – 420 nm. Count median diameters (CMD) and geometric standard deviations (GSD) were averaged and given as mean ± SD in **Table 2**. In addition, the provided SMPS parameters, volume median diameters (VMD) as well as number concentrations and volume concentrations, were averaged as well. Since the measured spectra of the SMPS were cut below 10 nm, spectral data down to a size of 1 nm were obtained by extrapolation of the averaged spectrum of 30 measured number size distribution spectra that was fitted to a lognormal size distribution minimizing the sum of least squares for the independent variables: median diameter, geometric standard deviation, and spectral peak height. Since the measured spectral data > 100 nm became noisy at the level below 10^-4^ of peak maximum, the fit was performed in the size range of 1 -100 nm. These corrections led to only minimally lower CMDs, GSD and VMDs (see Table 2). Averaged spectra of all groups of rats are presented in **Fig. S1B**; also the time course of the median diameters, the number concentrations during the inhalation exposure of each group of rats are shown in **Fig. S1C+S1D** of Supplementary Information. For S/TEM image analyses non-radio-labeled AgNP aerosols were produced using the same characteristic generation parameters as for the radiolabeled aerosols but using non-irradiated silver electrodes; a representative S/TEM image (Tecnai F20, FEI – Thermo Fisher Scientific, Waltham, ) are shown in **Figs. S1A + S1B.**

**Figure S1:** **A:** S/TEM image of AgNP (grand view) without radio-labeling using the same aerosol generation conditions as those including radio-labeling; the AgNP aerosols were generated under identical experimental conditions. **B:** S/TEM image of AgNP ( Magn. 200 000x, without radio-labeling, using the same aerosol generation conditions as those including radio-labeling) showing the granular structure of primary Ag-particles within each of several agglomerated AgNP. **C:** Mean spectra averaged over the original, 30 sequential [^105^Ag]AgNP aerosol size distribution SMPS spectra during exposure of each group of rats, respectively. Since the values of all channels > 100 nm showed Zero values we cut those out to provide a better resolution of the data between 10 and 100 nm. **D:** Median [^105^Ag]AgNP aerosol diameter of each of the 30 sequential SMPS spectra during the exposure of each group of rats, respectively. **D:** [^105^Ag]AgNP aerosol number concentration of each of the 30 sequential SMPS spectra during the exposure of each group of rats, respectively.

## In vitro dissolution of [^105^Ag]AgNP submersed in distilled water

In order to prove a common dissolution pattern of the spark generated [^105^Ag]AgNP similar to those reported in previous studies, cut sections of a [^105^Ag]AgNP aerosol filter (50 mm diameter, 0.1 µm pore size, PTFE) collected during the inhalation exposure of the group of rats to be dissected after 24 hours were used in triplicate. These filter sections were tightly covered at top and bottom with plain sandwich filters (50 mm diameter, 0.02 µm pore size, PTFE) in an annular filter holder and submersed in 10 ml distilled water in a Petri dish (60 mm diameter) at room temperature. After incubation times of 15 minutes, 1 hour, 1 day and 3 days the solvent was carefully pipetted off and the [^105^Ag]Ag activity of the solvent was measured γ-spectrometrically. The particulate fraction was determined from the sum of activities of the Petri dish together with the remaining piece of the filter and the [^105^Ag]Ag activity on a 10 kDa Amicon centrifugation filter used to determine the particulate fraction which was removed eventually with the solvent during pipetting. The results are shown in **Fig. S2**. The dissolved fraction amounts to already 0.15 after 15 minutes and increases to 0.33 after 1 hour without further increase of dissolution during 3 days of incubation. The partial [^105^Ag]AgNP dissolution is in agreement with previous studies[[1](#_ENREF_1)] resulting from the oxidative dissolution and depends on several parameters such as temperature, Ag-ion concentration, oxygen availability as shown earlier[[2](#_ENREF_2)].

**Figure S2:** Dissolution pattern of spark generated [^105^Ag]AgNP incubated in 10 ml distilled water up to 3 days. The [^105^Ag]AgNP had been collected on an aerosol filter during the inhalation exposure of the group of rats to be dissected after 24 hours. Tests were performed in triplicate.

## Intratracheal inhalation exposure to the freshly generated [^105^Ag]AgNP aerosol

As described earlier [[3](#_ENREF_3), [4](#_ENREF_4)], the four rats of each group were anesthetized by an intramuscular injection of a mixture of Medetomidin (15 *μ*g/100 g body weight), Midazolam (0.2 mg/100 g body weight), and Fentanyl (0.5 *μ*g/100 g body weight). For endotracheal intubation of the healthy, female, adult rats, a flexible cannula (16 G, 1.7 mm diameter, 50 mm length) was placed in the upper trachea under visual control and sealed against outside air with a modified pipette tip wedged gently into the laryngeal opening [[3-5](#_ENREF_3)]. Each rat was placed on its left lateral side in one of the four temperature-controlled plethysmographs (50 mm diameter, 20 cm length, volume 390 cm³, inside temperature 32°C) of the inhalation apparatus and the endotracheal cannula was connected with the aerosol line outside in front of the plethysmograph. Ventilation was computer-controlled with a negative pressure of −1.5 kPa applied to the plethysmograph for 0.3 s of inspiration followed by 0.3 s spontaneous expiration at ambient air pressure, resulting in a breathing frequency of 100 min^−1^. This ventilation pattern caused inspiration of about 75% of total rat lung capacity (TLC), so animals were slightly hyperventilated and did not breathe spontaneously but followed the computer-controlled breathing pattern, **Fig. S3**.

After the intratracheal inhalation, anesthesia of each rat was antagonized by subcutaneous injection of a mixture of Atipamezol (0.075 mg/100 g body weight), Flumazenil (20*μ*g/100 g body weight), and Naloxon (10 *μ*g/100 g body weight) as described earlier [[3](#_ENREF_3), [4](#_ENREF_4), [6](#_ENREF_6)].

- 1500 Pa

O

2

N

2

filter

sample

Ar

Ag

Ag

spark

generator

CPC

DMPS

-30 Pa

HUM.

Control

anesthetized

intubated rats

ventilated in

plethysmographs

Kr-85

600°C, tube

furnace

^105^Ag labeled

HEPA filtered secondary containment for radiation protection

Cu-tube

**Figure S3**: Schematics of the spark ignition aerosol generator and the aerosol inhalation apparatus taken from Supplementary Information of [[7](#_ENREF_7)]. DMPS: Differential Mobility Particle Sizer; CPC: Condensation Particle Counter; HUM: Humidifier. (2-column fitting image)

## Radiometric and statistical analysis

The [^105^Ag]Ag radioactivity of all samples was measured by γ-ray spectrometry without any further physico-chemical sample preparation in either a lead-shielded 10-mL or a lead-shielded 1-L well-type NaI(Tl) scintillation detector as previously described [[4](#_ENREF_4), [8-10](#_ENREF_8)]. The radioactive ^105^Ag isotope decays to ^105^Pd *via* electron capture and positron emission, with a half-life of 41.3 days; the 280 keV γ-ray emission line was used for the radiometric analysis. The count rates were corrected for physical decay and background radiation. Additionally, count rates were calibrated to a ^105^Ag reference source ([^105^Ag]AgNP filter sample of 2200 Bq (at reference date)) whose activity was determined by an absolutely calibrated Ge/Li semiconductor detector in order to correlate [^105^Ag]Ag-radioactivity to the mass of the AgNP making use of the specific ^105^Ag-radioactivity per Ag mass of 2.60 MBq/mg determined for the activated Ag-electrode tips. Samples yielding net counts (*i.e.*, background-corrected counts) in the 280 keV region-of-interest of the ^105^Ag γ-ray spectrum were considered below the detection limit when they were less than three standard deviations of the 200-minute background count rate in this region-of-interest. The background count rate was determined without any sample in the detector well. The detection limit (DL) corresponded to 0.1 Bq.

For a complete balance of the deposited [^105^Ag]Ag-radioactivity within the lungs of each rat, the [^105^Ag]Ag-activities of all individual samples (including those taken at the dissection time point and those during excreta collection) were summed up for each rat and the sum was used as a denominator for the calculation of the [^105^Ag]Ag-activity fraction in each sample per Initial Lung Dose (ILD). MCC was determined from the measured [^105^Ag]Ag-activity in samples of the gastro-intestinal-tract (GIT) and fecal excreta during the first days p.e. Note, MCC data do not contribute to the slow, long-term AgNP clearance processes from the peripheral lungs, like translocation across the ABB, long-term transport from the lung periphery towards the larynx, etc. The fractions for each organ or tissue were averaged over the four rats of each group and are reported with the standard error of the mean (SEM) as described earlier [[3](#_ENREF_3), [4](#_ENREF_4), [8-10](#_ENREF_8)].

All calculated significances are based on a One-Way-ANOVA test and a *post hoc* Bonferroni Test. In the case of an individual two-group comparison, the unpaired t-test was used. Significance was considered at p ≤ 0.05.

## Parameters of inhalation and deposition

In the following we use the index i to distinguish individual rats that are subjected to inhalation exposure of [^105^Ag]AgNP for a duration of 1½ hours in a group of animals labeled by the index j that were sacrificed after a certain retention period ("immediately after inhalation" (0.75), 4h, 24h, 7d, and 28d).

During each inhalation exposure of a group j of rats, an [^105^Ag]AgNP aerosol sample was collected on a filter, and the [^105^Ag]Ag-activity $A_{\text{filt}}^{\text{j}}$ (Bq) on the filter was determined by γ-spectrometry. The aerosol volume $V_{\text{filt}}^{\text{j}}$ (L) was measured with a gas flow meter and the specific aerosol activity $a_{\text{filt}}^{\text{j}}$ (Bq/L) was calculated as

|  | $a_{\text{filt}}^{\text{j}}=A_{\text{filt}}^{\text{j}}/V_{\text{filt}}^{\text{j}}$ | $\text{j}=1,..7$ | (**1**) |
| --- | --- | --- | --- |

The mean total lung capacity (TLC) in mL of healthy, female WKY-Kyoto Wistar rats was determined by [[11](#_ENREF_11)] to be$TLC=0.047\cdot BW$ (*BW*= body weight in gram). At a ventilation-pressure of -1.5 kPa, the tidal volume $TV_{\text{i}}^{\text{j}}$ of each intubated rat i of group j was set at 75% *TLC*, which gives

|  | $TV_{\text{i}}^{\text{j}}=0.75\cdot TLC_{\text{i}}^{\text{j}}=0.75\cdot0.047\cdot BW_{\text{i}}^{\text{j}}$ | $i=1,2,3,4 rats; j=1,..7 group$ | (**2**) |
| --- | --- | --- | --- |

The rats were ventilated for a ventilation time $t_{\text{inhal}}$ of 1½ hours at a ventilation rate $f_{\text{i}}^{\text{j}}$ of 100 min^-1^ set by the computer-controlled inhalation apparatus. The inhaled aerosol volume $V_{inhal,i}^{j}$ of each rat i of group j was calculated as

|  | $V_{\text{inhal,i}}^{\text{j}}=f_{\text{i}}^{\text{j}}\cdot TV_{\text{i}}^{\text{j}}\cdot t_{\text{inhal}}$ | $i=1,2,3,4 rats; j=1,..7 group$ | (**3**) |
| --- | --- | --- | --- |

## Preparation of biokinetics samples for radiometric analysis

At the chosen retention times of "immediately after inhalation" (0.75h), 4h, 24h, 7d or 28d after a 1½

-hour [^105^Ag]AgNP intratracheal inhalation, rats were anesthetized (by 5 % isoflurane inhalation) and euthanized by exsanguination via the abdominal aorta as described earlier [[3](#_ENREF_3), [4](#_ENREF_4), [8-10](#_ENREF_8)]. Note, the immediate time point after the 1½-hour inhalation is set to be the 0.75h time point. Approximately 0.7 of the total fractional blood volume could be recovered. Total organs, tissues, the entire remaining carcass, and total fecal and urinary excretions were collected for radiometric analysis (see **Table S2**). During dissection, none of the organs were cut and all fluids were cannulated (where necessary) in order to avoid any cross-contamination. In addition, broncho-alveolar lavages (BAL) were performed as described in the Methods section of the main paper. Organs, tissues, carcass, BAL and excretions were collected for radio-analysis (see **Table S2**).

**Table S2:** Total organs, tissue and other samples prepared for radiometric analysis:

| lungs* | trachea + main bronchi, incl. hilar lymph nodes | | BAL cells* | BAL fluid* |
| --- | --- | --- | --- | --- |
| liver_(2nd)_ | spleen_(2nd)_ | | kidneys_(2nd)_ |  |
| brain_(2nd)_ | heart_(2nd)_ | | uterus_(2nd)_ |  |
| GIT: gastrointestinal tract, comprising the esophagus, stomach, small and large intestine | | | | |
| total skin_(2nd)_ | muscle sample_(2nd)_ | skinned head_(2nd)_  without brain | | |
| exsanguinated blood sample_(2nd)_ | | | | |
| carcass_(2nd)_: total remaining carcass beyond the listed organs and tissues including the skeleton and soft tissue but excluding the skin | | | | |
| Secondary organs_(2nd)_: the sum of all secondary organs indexed with _(2nd)_. | | | | |
| excretion: total daily urine and feces, collected separately; in the 28-day group integral urinary and fecal excretion was collected over 3-4 days as described in the Methods section of the main paper. | | | | |

* Lavaged lungs; broncho-alveolar lavage (BAL); separation of BAL cells from BAL fluid supernatant by centrifugation as described in [[12](#_ENREF_12)]

(2nd) The index “_(2nd)_“ indicates those secondary organs and tissues in which [^105^Ag]AgNP may have accumulated after translocation across the air-blood-barrier (ABB) into blood circulation.

## Blood correction and total blood volume

In order to obtain the true value of [^105^Ag]Ag-activity in the organs and tissues of interest, the radioactivity contributed by the residual blood retained after exsanguination had to be subtracted.

**Table S3:** Organ-specific weight factors $f_{\text{organ,} \text{i}}^{\text{Oeff}}$ for the residual blood in the organ tissue after exsanguinations, given as residual blood weight per organ weight according to [[13](#_ENREF_13)].

| Lung (g•g^-1^) | liver (g•g^-1^) | spleen (g•g^-1^) | kidney (g•g^-1^) | brain (g•g^-1^) |
| --- | --- | --- | --- | --- |
| 0.28 ± 0.10) | 0.14 ± 0.03 | 0.16 ± 0.04 | 0.22 ± 0.04 | 0.018 ± 0.001 |
| heart (g•g^-1^) | GIT^§^  (g•g^-1^) | muscle (g•g^-1^) | fat (g•g^-1^) | thyroid* g/organ |
| 0.15 ± 0.02 | 0.020 ± 0.006 | 0.016 ± 0.002 | 0.012 ± 0.002 | 0.008 ± 0.001 |

* residual blood for the thyroid is given for complete organ; ^§^ gastrointestinal tract

The blood contents of organs and tissues were calculated according to the findings of Oeff and Konig [[13](#_ENREF_13)] shown in **Table S3** and the [^105^Ag]Ag-radioactivities of the organs were corrected for these values as

|  | $A_{\text{organ}_{\text{ }\text{k}}\text{, i}}^{\text{j}}=A_{\text{organ}_{\text{ }\text{k}}\text{, i}}^{\text{measured, j}}-A_{{\text{organ}\text{ }}_{\text{k}}\text{, i}}^{\text{blood correction, j}}$ | $i=1,2,3,4 rats; j=1,..7 group$ | (**4**) |
| --- | --- | --- | --- |

where $A_{\text{organ}_{\text{k}}\text{,i}}^{\text{measured}\text{, j}}$ denotes the [^105^Ag]Ag-activity measured in the organ "k" of rat _i,j_ ($i=1,2,3,4 rats; j=1,...7\mathrm{group}$ for retention times of 0.75h, 4h, 24h, 7d, and 28d) in (Bq) which is corrected for the residual blood content by subtracting $A_{\text{organ}_{\text{k}}\text{, i}}^{\text{blood correction, j}}$ calculated according to

|  | $A_{\text{organ}_{\text{k}}\text{,i}}^{\text{blood correction, j}}=\frac{A_{\text{blood sample, i}}^{\text{measured, j}}\cdot m_{\text{organ}_{\text{k}}\text{,i}}^{\text{measured, j}}\cdot f_{\text{organ}_{\text{k}}}^{\text{Oeff}}}{m_{\text{blood sample, i}}^{\text{measured, j}}}$ | $i=1,2,3,4 rats;$ $j=1,..7 group$ | (**5**) |
| --- | --- | --- | --- |

making use of the mass $m_{\text{blood sample, i}}^{\text{measured, j}}$ and the [^105^Ag]Ag-activity $A_{\text{blood sample, i}}^{\text{measured, j}}$ measured for the blood recovered from exsanguination, the mass of the organ $m_{\text{organ}_{\text{k}},i}^{\text{measured, j}}$ and the organ-specific weight factor $f_{\text{organ}_{\text{k}}}^{\text{Oeff}}$ of **Table S3** for the residual blood in the organ tissue according to Oeff and Koenig (1955)[[13](#_ENREF_13)]. The total blood volume *BV* in mL was estimated to be

|  | $BV=0.06\cdot BW+0.77$ |  | (**6**) |
| --- | --- | --- | --- |

according to the work of Lee and Blaufox [[14](#_ENREF_14)], where *BW* denotes the body weight in g.

To determine the [^105^Ag]Ag-activity in the residual blood of the remaining carcass for each rat the following procedure was applied. Firstly, the mass of the residual blood volume in the carcass $m_{\mathrm{carc}_{\text{k}}\text{, i}}^{\text{res.-blood, j}}$ was calculated by subtracting the mass of the sampled blood volume $m_{\text{meas.-blood sample, i}}^{\text{ j}}$from the mass of the total blood volume $m_{BV}$ and the sum of the masses of the residual blood volumes of all organs $m_{\text{organ}_{\text{k}}\text{, i}}^{\text{res.-blood, j}}$ according to [[13](#_ENREF_13)]

|  | $m_{\text{carc}_{\text{k}}\text{, i}}^{\text{res.-blood, j}}=m_{\mathrm{BV}}-m_{\text{meas.-blood sample, i}}^{\text{j}}-\sum_{\text{l}\neq\text{k}} {m_{\text{organ}_{\text{l}}\text{,i}}^{j}}\cdot f_{\mathrm{org}_{\text{l}}\text{,i}}^{\text{Oeff}}$ | $i=1,2,3,4 rats;$ $j=1,..7 group$ | (**7**) |
| --- | --- | --- | --- |

For each rat, the [^105^Ag]Ag-activity in the residual blood of the remaining carcass $A_{\text{carc}_{\text{k}}}^{\text{res.-}\text{blood }}(i,j)$ is then given by:

|  | $A_{\text{carc}_{\text{k}}\text{, i}}^{\text{res.-blood, j }}=\frac{A_{\text{meas.-blood sample, i}}^{\text{j}}}{m_{\text{meas.-blood sample, i}}^{\text{j}}}\cdot m_{\text{carc}_{\text{k}}\text{, i}}^{\text{res.-blood, j}}\cdot\frac{{m_{\text{carc}_{\text{k}}\text{, i}}^{j}}}{m_{\text{BW, i}}^{j}}$ | $i=1,2,3,4 rats;$ $j=1..5 group$ | (**8**) |
| --- | --- | --- | --- |

This estimate assumes that the residual blood volume is proportional to the mass of the carcass.

To determine the contribution of the [^105^Ag]Ag-activity in the residual blood relative to the total [^105^Ag]Ag-activity retained in all organs and tissues, the ratio $RB_{\text{organ(k), i}}^{\text{blood correction, j}}$ is defined:

|  | $RB_{\text{organ}_{\text{k}}\text{, i}}^{\text{blood correction, j}}=\frac{A_{\text{organ}_{\text{k}}\text{, i}}^{\text{blood correction, j}}}{A_{\text{meas.-organ}_{\text{k}}\text{, i}}^{\text{j}}}$ | $i=1,2,3,4 rats; j=1,..7 group$ | (**9**) |
| --- | --- | --- | --- |

Where the [^105^Ag]Ag-activity of the residual blood retained in each organ,$A_{\text{organ(k), i}}^{\text{blood correction, j}}$, is calculated according to **Eqn.** (**5**). As the retention of [^105^Ag]Ag-activity in blood, organs, and tissues depends on time and follows different patterns, the ratio $RB_{\text{organ(k), i}}^{\text{blood correction, j}}$ itself depends on time. This is shown in **Fig. 8** for all organs and the remaining carcass.

The ratios in **Fig. 8** are larger than 0.1 during the first 24-hours p.e. for most of the secondary organs and somewhat smaller in the carcass (comprising soft tissue and skeleton). This may have resulted from initially rather high amounts of translocated and still circulating [^105^Ag]AgNP and their degradation products in blood and retarded accumulations in the secondary organs and carcass. But corrections are very small in the (lavaged) lungs since the dominant part of the [^105^Ag]AgNP was retained in the lungs and the [^105^Ag]Ag-activity concentration in blood was very low.

## Total [^105^Ag]AgNP deposition in each rat determined by the balanced [^105^Ag]Ag activities of the entire dissected rat including its total excretions

The inhaled aerosol activity$A_{\text{inhal,i}}^{\text{j}}$ is calculated from the product of the total inhaled volume $V_{\text{inhal,i}}^{\text{j}}$ determined from **Eqn.** (**3**) and the specific [^105^Ag]Ag aerosol activity $a_{\text{filt}}^{\text{j}}$ ((Bq/L) (**Eqn.** (**1**)) as

|  | $A_{\text{inhal,}\text{ }\text{i}}^{\text{j}}=V_{\text{inhal,}\text{ }\text{i}}^{\text{j}}\cdot a_{\text{filt}}^{\text{j}}$ | $i=1,2,3,4 rats; j=1,..7 group$ | (**10**) |
| --- | --- | --- | --- |

In order to determine the initially deposited [^105^Ag]AgNP activity $A\text{(ILD)}_{\text{i}}^{\text{j}}$ (Bq) in the lungs of each rat (*i.e.* ILD), which is smaller than the inhaled dose because a significant amount of nanoparticles are exhaled again, all γ-spectrometrically measured sample activities $A_{\text{sample k,i}}^{\text{j}}$were summed up, where the index k identifies all specimens (all samples of organs, tissues, total excretion of rat (i,j)) collected from a single animal i of group j according to

|  | $A{(\text{ILD})}_{\text{i}}^{\text{j}}=\sum_{\text{k}=1}^{\text{n}} A_{\text{sample k,}\text{ }\text{i}}^{\text{j}}$ | k = 1..n; all samples of organs, tissues, total excretion of rat (i,j) $i=1,2,3,4 rats; j=1,..7 group$ | (**11)** |
| --- | --- | --- | --- |

For the total deposited [^105^Ag]AgNP mass $m\text{(ILD)}_{\text{i}}^{\text{j}}$ (µg) and [^105^Ag]AgNP number $N\text{(ILD)}_{\text{i}}^{\text{j}}$ the total deposited [^105^Ag]AgNP activity $A\text{(ILD)}_{\text{i}}^{\text{j}}$ of each rat was divided by the specific [^105^Ag]Ag activity concentration per [^105^Ag]Ag-mass (2.60 kBq/µg) or divided by the quotient of the specific aerosol activity $a_{\text{filt}}^{\text{j}}$(Eqn. **1**) and the averaged aerosol number concentration $N\text{(Aer)}_{\text{i}}^{\text{j}}$ (**Table 1**) determined by the CPC 3022A, respectively.

|  | ${m(\mathrm{ILD})}_{i}^{j}= {A(\mathrm{ILD})}_{i}^{j} \times2.60 (\frac{\mathrm{MBq}}{\mathrm{mg}})$ $N(\text{ILD})_{\text{i}}^{\text{j}}=N(\text{Aer})_{\text{i}}^{\text{j}}\frac{A(\text{ILD)}_{\text{i}}^{\text{j}}}{a_{\text{filt, i}}^{\text{j}}}$ | $i=1,2,3,4 rats; j=1,..7 group$ | (**12a**)  (**12b**) |
| --- | --- | --- | --- |

In addition, the deposited [^105^Ag]AgNP activity fraction $a\text{(ILD) }_{\text{i}}^{\text{j}}$ was determined relative to the total inhaled aerosol activity of each rat according to Eqn. (**10**).

|  | $a\text{(ILD)}_{\text{i}}^{\text{j}}=\frac{A\text{(ILD)}_{\text{i}}^{\text{j}}}{V_{\text{inhal,i}}^{\text{j}}\cdot a_{\text{filt}}^{\text{j}}}$ | $i=1,2,3,4 rats; j=1,..7 group$ | (**13**) |
| --- | --- | --- | --- |

In the next step [^105^Ag]AgNP activity fractions in organs and tissues (index k) $a\text{(ILD)}_{\text{sample k,i}}^{\text{j}}$with respect to the deposited dose in the lungs were calculated by normalizing all measured sample activities $A_{\text{sample k,}\text{ }\text{i}}^{\text{j}}$ (Bq) to the total deposited [^105^Ag]Ag-activity $A\text{(ILD)}_{\text{i}}^{\text{j}}$ (Bq) of each rat (i,j) according to

|  | $a{(\text{ILD)}}_{\text{sample k,}\text{ }\text{i}}^{\text{j}}=\frac{A_{\text{sample k,}\text{ }\text{i}}^{\text{j}}}{A\text{(ILD)}_{\text{i}}^{\text{j}}}$ | $i=1,2,3,4 rats; j=1,..7 group$ k = 1..n; all samples of organs, tissues, total excretion of rat (i,j) | (**14**) |
| --- | --- | --- | --- |

In case of the partially soluble [^105^Ag]AgNP, mucociliary clearance (MCC) from the conducting airway epithelium towards the larynx and into the GIT is superimposed by rapid translocation of precipitates of nano-sized silver salt clusters consisting of poorly soluble [^105^Ag]AgCl_3_, [^105^Ag]Ag_2_S, [^105^Ag]Ag_2_Se, [^105^Ag]AgPO_4_ [[15](#_ENREF_15)] [[16](#_ENREF_16)] [[17](#_ENREF_17)] which are rapidly formed in the ion-rich epithelial lining fluid by [^105^Ag]Ag^+^ ions dissolving from the surface of the [^105^Ag]AgNP (see the discussed literature in the Background section of the main paper). The precipitated silver salt clusters may translocate rapidly across the lung epithelium into blood towards the GIT via liver and gall bladder. Hence, the early clearance data during the first 24 h p.e. comprise both clearance pathways which need to be considered: translocation across the ABB into the blood and via liver and gall bladder into the GIT as well as mucociliary clearance to the larynx and into the GIT. After IT instillation, the dominant amount of ^110m^Ag activity has already reached the hindgut or is fecally excreted (as described in the Results section of the main paper). Hence, quantitative discrimination of the mucociliary clearance *versus* the ABB translocation to blood is not directly possible after IT instillation or IV injection of soluble [^110m^Ag]AgNO_3_. Furthermore, after the inhalation of the 20-nm-sized [^105^Ag]AgNP both particle species compete for MCC clearance not allowing a direct estimate of the possible contribution of either species.

In the following equation the calculation of MCC of [^105^Ag]AgNP and their degradation products are given:

|  | $A\text{(ILD) }_{\text{MCC,}\text{ }\text{i}}^{\text{j}}=A\text{(ILD) }_{\text{head}-\text{brain,}\text{ }\text{i}}^{\text{j}}+A\text{(ILD) }_{\text{trachea,}\text{ }\text{i}}^{\text{j}}+A\text{(ILD) }_{\text{GIT,}\text{ }\text{i}}^{\text{j}}+A\text{(ILD) }_{\text{feces,}\text{ }\text{i}}^{\text{j}}$ | | (**15**) | |
| --- | --- | --- | --- | --- |
|  | $a\text{(ILD)}_{\text{MCC,}\text{ }\text{i}}^{\text{j}}=\frac{A\text{(ILD)}_{\text{MCC,}\text{ }\text{i}}^{\text{j}}}{A\text{(ILD)}_{\text{i}}^{\text{j}}}$ | $i=1,2,3,4 rats; j=1,..7 group$ | | (**16**) |

Where $A\text{(ILD) }_{\text{head}-\text{brain,}\text{ }\text{i}}^{\text{j}}$ denotes the [^105^Ag]Ag-activity determined for the head without the brain, $A\text{(ILD) }_{\text{trachea,}\text{ }\text{i}}^{\text{j}}$ the [^105^Ag]Ag-activity in the trachea, $A\text{(ILD) }_{\text{GIT,}\text{ }\text{i}}^{\text{j}}$ the [^105^Ag]Ag-activity in the gastrointestinal tract and $A\text{(ILD) }_{\text{feces,}\text{ }\text{i}}^{\text{j}}$ the activity of the feces collected during the first 48h p.e.

Quantitative discrimination of the slow long-term clearance (LTC) versus the ABB translocation to blood is not directly possible after IT instillation or IV injection of soluble [^110m^Ag]AgNO_3_. As described above for MCC, after the inhalation of the 20-nm-sized [^105^Ag]AgNP both particulate species compete for LTC clearance not allowing a direct estimate of the possible contribution of either species. To estimate the long-term cleared [^105^Ag]AgNP fraction (LTC) from the alveolar epithelium via the ciliated conducting airways towards the larynx for subsequent swallowing into the GIT and fecal excretion, the total fecal excretion after day 2 is calculated by summing up all fecal samples from day 3 up to day m (d_m_).

|  | $\tilde{a}_{\text{LTC,i}}^{\text{j}}=\sum_{\text{l}=3}^{m} a_{\text{feces l,}\text{ }\text{i}}^{\text{j}}$ | l = 3,…m; d_m_ ∈ [3,…28 day] $i=1,2,3,4 rats; j=1,..7 group$ | (**17**) |
| --- | --- | --- | --- |

## Lung and body retention fits

The lung retention LR(t) in **Fig. S4A** is based on the data obtained from the five dissection time points (Table 3: 1^st^ line of “total lungs”) which were fitted to a two-term, exponential function using a least-squares algorithm resulting in:

|  | $LR\left( t \right)=0.83 \times\exp\left\{ - \frac{\ln2 \times t}{2.5 d} \right\} +0.22 \times\exp\left\{ -\frac{\ln2 \times t}{55 d} \right\}$ | t ∊ [3, 28 d] | (**18a**) |
| --- | --- | --- | --- |

For comparison, the body retention, BR(t) of the 28d group of rats, in **Fig. S4B** is dominated by the lung retention with minor contributions of the translocated fractions that are present in all secondary organs, tissues, and blood (see Table 3). It can be estimated from the excretion data determined between day 3 and day 28 according to:

|  | $BR(t)=1-\bar{a}_{\text{feces,cumul}}(t)-\bar{a}_{\text{urine,cumul}}(t)$ | t ∊ [3, 28 d] | (**18b**) |
| --- | --- | --- | --- |

where $\bar{a}_{\text{feces,cumul}}(t)$and $\bar{a}_{\text{urine,cumul}}(t)$ denote the mean cumulative activity fractions of the 28d group of rats excreted up to time $t$ via feces and urine, respectively. A least-square fit of the body retention to a two-term-exponential function gives

|  | $BR\left( t \right)=1.06 \times\exp\left\{ -\frac{\ln2 \times t}{1.63 d} \right\}+0.18 \times\exp\left\{ -\frac{\ln2 \times t}{19.3 d} \right\}$ | t ∊ [1, 28 d] | (**18c**) |
| --- | --- | --- | --- |

**Figure S4**: **A:** Mean lung retained fractions (per ILD) obtained from five dissection time points (open squares, data taken from **Table 4**); half-lives are 2.5 and 55 d. **B:** Mean body retention of the 28-day group of rats from day 3 to day 28 according to Eqn. (**18c**) (upright open triangles) together with least squares using a bi-exponential fit; half-lives are 1.6 days and 19 d, respectively. Additionally, the body retention of the 7-days group (open circles) is shown. (2-column fitting image)

The steep decline of lung retention cannot be fitted by a mono-exponential term. While in **Fig. S4A** the fitted long-term exponential term is essentially determined by three time-points – days 1, 7 and 28 – the fitted half-life of 55 d can only be a rough estimate. In contrast, body retention in **Fig. S4B** – derived according to **Eqn. 18C** – based on more data points may be more realistic.

|  | ${BR\left( t \right)}_{i}^{j}= {A(\mathrm{ILD})}_{i}^{j}- \int A_{fec, i}^{j}(t) dt - \int A_{urine, i}^{j}(t) dt$ | $i=1,2,3,4 rats;$ $j=1,..7 group$ t ∊ [1, 28 d] | (**19**) |
| --- | --- | --- | --- |

Both fitted half-lives of the bi-exponential term – 1.6 and 19 d, respectively, - are shorter than those of the measured lung data. The body retention data between day 3 and day 7 of the 28d group of rats are very well confirmed by those of the 7d group of rats (**Fig. S4B**).

The deposition fractions obtained from the intratracheally inhaled (*i.e.* low-pressure ventilated and endotracheally intubated) rats – given in **Table 2** - were compared with the estimates by the MPPD Software (vs. 3.04) [[18](#_ENREF_18)] shown in **Fig. S5**. In **Table S1** the input parameters used for the MPPD modeling are compiled. The aerosol parameters are adjusted to the exposure conditions of the present study given in **Table 2**.

**Figure S5**: Total and regional deposition fractions in adult rats estimated by the MPPD Software (vs. 3.04) according to endotracheal breathing. All MPPD model parameters are listed in **Table S1**; TB – deposited fraction in the tracheobronchial region, Pulm - deposited fraction in the pulmonary region. (2-column fitting image)

**Table S1:** Model parameters used in the MPPD simulation.

|  | |  | **MPPD model (version 3.04)** | |  |  |
| --- | --- | --- | --- | --- | --- | --- |
| **Species & Model** | | | **Physiology parameters** | | **aerosol parameters** | |
| species | adult Long Evans rat | | TLC (cm³) | 13.7 | CMD (µm) | 0.022 |
| geometry | assym. mult. path | | FRC (cm³) | 4.0 | GSD | 1.4 |
| exposure | endotracheal | | breath frequency (min^-1^) | 100 | density (g cm^-3^) | 4.7 |
| position | left lateral | | TV (cm³) | 4.0 | aspect ratio | 1 |
|  |  | | time of inspiration (sec) | 0.3 | aerosol concen-tration (mg m^-3^) | 2.5 |
|  |  | | pause (sec) | 0 |  |  |

## Bronchoalveolar lavage (BAL)

Serial broncho-alveolar lavages (BAL) of rat lungs were performed at each time point of dissection. As discussed above the [^105^Ag]AgNP retention on the lung epithelium versus free [^105^Ag]AgNP suspended in the BAL-fluid, versus [^105^Ag]AgNP associated with freely moving lung surface macrophages was superimposed by the dissolution kinetics of released [^105^Ag]Ag-ions from the surface of [^105^Ag]AgNP leading to precipitating [^105^Ag]Ag–salt clusters contributing to the particle-related clearance mechanisms.

BALs were performed by applying 6 x 5 ml of phosphate-buffered-saline solution (PBS without Ca^2+^ or Mg^2+^) under the gentle massage of the thorax. The recovered BAL fluid (BALF) fraction (about 0.8 of instilled PBS) was centrifuged at 500 g for 20 min at room temperature to separate the lavaged cells from the supernatant. The [^105^Ag]AgNP content was determined by γ-ray-spectrometry.

By γ-ray-spectrometry, the fractions of free [^105^Ag]AgNP and their poorly soluble degradation products in the BALF and those particulates in BAL cells and in the lavaged lungs were determined. Applying this BAL procedure we obtained about 6 • 10^6^ macrophages per BAL which is on average very similar to our previous studies [[3](#_ENREF_3), [4](#_ENREF_4), [19](#_ENREF_19)]. Normalizing the number of totally recovered macrophages of each BAL to the mean number of the total surface macrophage population of (12.5 ± 0.8) • 10^6^, previously determined in the lungs of WKY rats [[19](#_ENREF_19)], we estimated the total fraction of [^105^Ag]AgNP and their degradation products associated with lung surface macrophages (alveolar macrophages (AM) pool) using the factor 0.5.

**Fig. S6** shows the latter and the sum of both particulate species of [^105^Ag]Ag in the lungs (persistent cores of [^105^Ag]AgNP and poorly soluble [^105^Ag]Ag-salt clusters) in the total lungs ${LU}_{tot,i}^{j}($t_j_$)$ and the lavaged lungs ${LU}_{lav,i}^{j}($t_j_$)$ beyond those particulate species in the AM pool $\hat{AMP}_{lav, i}^{j}\left( t_{j} \right)$and also [^105^Ag]Ag in BAL fluid ${BALF}_{i}^{j}(t_{j})$. Note that the fractions in the lavaged lungs are normalized to the contemporary lung burden as determined by:

|  | ${LU}_{tot, i}^{j}(t_{j})={LU}_{lav, i}^{j}(t_{j})+ {BALC}_{i}^{j}(t_{j})+{BALF}_{i}^{j}(t_{j})$ Normalization to contemporary lung burden ${LU}_{\mathrm{tot}, i}^{j}(t_{j})$: $\hat{LU}_{lav, i}^{j}\left( t_{j} \right)= \frac{{LU}_{lav, i}^{j}(t_{j})}{{LU}_{tot, i}^{j}(t_{j})}$ ; $\hat{BALC}_{lav, i}^{j}\left( t_{j} \right)= \frac{{BALC}_{lav, i}^{j}(t_{j})}{{LU}_{tot, i}^{j}(t_{j})}$ ; $\hat{BALF}_{lav, i}^{j}\left( t_{j} \right)= \frac{{BALF}_{lav, i}^{j}(t_{j})}{{LU}_{tot, i}^{j}(t_{j})}$ And for the AM pool $\hat{AMP}_{lav, i}^{j}\left( t_{j} \right)$ $\hat{AMP}_{\mathrm{lav}, i}^{j}\left( t_{j} \right)= \frac{1}{eff} \times\hat{BALC}_{lav, i}^{j}\left( t_{j} \right)$ | $i =1,2,3,4 rats;$ $j=1,..7 group$ $t_{j} \in\left[ 1h, \ldots28d \right]$ $eff=0.5$ BAL efficiency | (**20**) |
| --- | --- | --- | --- |

In contrast to **Fig. 2** of the main paper – which showed the rapidly declining [^105^Ag]Ag in the lungs – the sum of both particulate species of [^105^Ag]Ag normalized to the contemporary lung burden highlights the quickly diminishing fractions of the particulate species the AM pool after 24h p.e. In fact, the data at day-7 and day-28 p.e. indicate that almost no particulate [^105^Ag]Ag species are retained in the AM pool but are relocated within the epithelial barrier and across in the lung interstitium and no re-entrainment from there into the AM-pool is detectable as observed after intratracheal inhalation of all three poorly soluble, 20-nm-sized [^195^Au]AuNP [[9](#_ENREF_9)], [^192^Ir]IrNP [[3](#_ENREF_3), [4](#_ENREF_4), [19](#_ENREF_19)] and [^48^V]TiO_2_-NP [[20](#_ENREF_20)]. Since the data in the AM pool are similar to those in BALF from 24 hours p.e. on, no differentiation between particulate and non-particulate [^105^Ag]Ag species are possible.

**Figure S6:** Estimated retention fractions of particulate [^105^Ag]Ag species for the total lung surface macrophage pool [[19](#_ENREF_19)] derived from the experimentally determined kinetics of lavageable particulate [^105^Ag]Ag species fractions associated with BALC (upward triangles) and the retained particulate [^105^Ag]Ag species in the lavaged lungs subtracted by the total lung surface macrophage pool (squares). Fractions associated with BALF may contain free particulate [^105^Ag]Ag species as well as soluble [^105^Ag]Ag fractions (cross). Fractions are normalized to the contemporary lung burden at 0.75 hours to 28 days after intratracheal inhalation derived from the exponential fit of **Eqn. 18A** and **Fig. S4**. Additionally, the fractions of free [^48^V]TiO_2_-NP in BALF are presented, which rapidly decline p.e. (crosses). Data points are mean ± SEM; n = 4 rats per time point.

## Estimated clearance rates of translocated [^105^Ag]Ag-salt clusters across the ABB into the blood circulation and GIT

As noted in the main paper we assume similar deposition probabilities of the inhaled 20 nm-sized AgNP and AuNP in the same strain of rats. In addition, we also assume a similar MCC kinetics of both NP and thereafter, a similar dynamics of the long-term clearance towards the larynx. Then we can estimate the contribution of the cleared fractions of the poorly soluble [^105^Ag]Ag-salt clusters crossing the ABB into blood and their elimination via liver and gall bladder into the GIT by subtracting the mean fractional fecal excretion rates obtained after inhalation of [^195^Au]AuNP, from the mean fractional fecal excretion rates after inhalation of [^105^Ag]AgNP shown in **Fig. 10 C+D**. This is performed for the respective 28d group of rats for both NP.

MCC is delayed due to the passage through the GIT but the same for both NP. Therefore, the differences of the mean fecal excretion rates can be estimated from the first day p.e. on until day 28.

Mean excretion rates of day-1 to day-28 $\left\{ \tilde{a}_{fec,i}^{\mathrm{NP}}\left( d_{j} \right) \right\}$ of the respective 28-day group of rats are:

|  | $\tilde{a}_{\mathrm{fec},i}^{\mathrm{Au}}(d_{j})= \sum_{i=1}^{4} {a_{fec,i}^{\mathrm{Au}}}(d_{j})$ $\tilde{a}_{\mathrm{fec},i}^{\mathrm{Ag}}(d_{j})= \sum_{i=1}^{4} {a_{fec,i}^{Ag}}(d_{j})$ | $i=1,..4 rats$; $d_{j}$∈[1,…28 day] after[^195^Au]AuNP inhalation  d_j_ $\in\left[ 1d, \ldots28d \right]$ | (**21**) |
| --- | --- | --- | --- |

Then the mean fractional clearance rates of [^105^Ag]Ag-salt clusters $\left\{ \tilde{a}_{\mathrm{clust}}^{Ag}\left( d_{j} \right) \right\}$ crossing the ABB into the blood and finally into the GIT are:

|  | $\tilde{a}_{\mathrm{clust}}^{Ag}\left( d_{j} \right)= \tilde{a}_{\mathrm{fec},i}^{\mathrm{Ag}}\left( d_{j} \right)- \tilde{a}_{\mathrm{fec},k}^{\mathrm{Au}}(d_{j})$ | $i=1,..4 rats$; $d_{j}$∈[1,…28 day]  k = 1,…4 rats of AuNP study [7] | (**22**) |
| --- | --- | --- | --- |

These rates are presented in **Fig. 10E** of the main paper.

## Accumulation and retention of [^105^Ag]AgNP and their poorly soluble degradation products in secondary organs and tissues relative to translocated [^105^Ag]AgNP across the ABB

In order to determine the amount of [^105^Ag]AgNP and their poorly soluble degradation products in secondary organs and tissues as a fraction of [^105^Ag]Ag activity that crossed the ABB, we normalize all activity values measured for all secondary organs and tissues $A_{\text{2nd organ, i }}^{\text{ j}}(t)$, all urine samples $A_{\text{urine, i }}^{\text{ j}}(t)$and all blood samples $A_{\text{blood} \text{sample, i}}^{\text{ j}}$to a value which is smaller than the value $A(t=0)=A_{0}$ that has been deposited after the 1½-hour intratracheal inhalation. The normalization activity $A_{\text{t-loc,i}}^{\text{j}}$ is the sum of all, $A_{\text{u}rine,\text{ i }}^{\text{ j}}(t)$,$A_{\text{blood} \text{sample, i }}^{\text{ j}}(t)$ . Therefore,

|  |  | $i=1,2,3,4 rats;$ $j=1,..7 group$ k=1,2..n; 2^nd^ organs  l=1,2..o; blood sample  m=1,2..p; urine sample | (**23**) |
| --- | --- | --- | --- |

holds for all rats i in each group j, which will be referred to as the translocated dose, which is a specific value for each rat used in the five retention time groups. The fractions of [^105^Ag]AgNP that have been accumulated in organs and tissues k, blood l, and urine m after passing the ABB can now be determined for each rat by normalizing the activities $A_{\text{organ}_{\text{k}}\text{, i}}^{j}$ according to

|  | $\hat{a}_{\text{organ}_{\text{k}}\text{,i}}^{\text{j}}=\frac{A_{\text{organ}_{\text{k}}\text{,i}}^{\text{j}}}{A_{\text{t-loc,i}}^{\text{j}}}$ $\hat{a}_{\text{blood}_{\text{l}}\text{,i}}^{\text{j}}=\frac{A_{\text{blood}_{\text{l}}\text{,i}}^{\text{j}}}{A_{\text{t-loc,i}}^{\text{j}}}$ $\hat{a}_{\text{urine}_{\text{m}}\text{,i}}^{\text{j}}=\frac{A_{\text{urine}_{\text{m}}\text{,i}}^{\text{j}}}{A_{\text{t-loc,i}}^{\text{j}}}$ | $i=1,2,3,4 rats; j=1,..7 group$ k=1,2..n; 2^nd^ organs  l=1,2..o; blood sample  m=1,2..p; urine sample | (**24**) |
| --- | --- | --- | --- |

## Auxiliary biokinetics studies after application of soluble [^110m^Ag]AgNO_3_ applications

**Fig. S7** shows the kinetics of fecal and urinary excretion rates after three applications of [^110m^Ag]AgNO_3_ solutions: intratracheal instillation (IT), intravenous injection (IV), oral instillation (GAVage). After IV and GAV application fractions of about 0.4 were fecally excreted at the end of the first day p.e. but only 0.25 after IT application indicating a slight delay of translocated [^110m^Ag]Ag across the ABB into the blood and subsequently via liver and gall bladder into the small intestine for fecal excretion. However after the first two days p.e. fecally excreted fractions after all three applications amount to 0.4. Thereafter, IT and IV applied [^110m^Ag]Ag decline in parallel to a fraction of 0.01 at day 7 p.e. while the fractions after GAV are more than tenfold lower (0.0003) after 7 days.

Urinary excretion rates are two orders of magnitude lower after IT and IV than fecal excretion rates indicating minimal filtration in renal glomeruli. Again the urinary excretion rates after IT and IV run in parallel while rates after GAV application are tenfold lower than those after IT and IV.

The kinetics after all three applications of [^110m^Ag]AgNO_3_ solutions confirm that the precipitation of nano-sized [^110m^Ag]Ag-salt clusters in the various body fluids leads to a prolonged elimination via the GIT into feces with a rapid early phase during the first two days p.e. followed by a gradually declining slow phase.

**Figure S7**: Kinetics of fecal (**panel A**) and urinary (**panel B**) excretion rates after intratracheal instillation (IT), intravenous injection (IV), oral instillation (GAVage) of [^110m^Ag]AgNO_3_ solutions. Data are given as mean ± SEM, n = 4 rats/time-point.

1. **References**

1. Loza K, Diendorf J, Sengstock C, Ruiz-Gonzalez L, Gonzalez-Calbet JM, Vallet-Regi M, Koller M, Epple M: **The dissolution and biological effects of silver nanoparticles in biological media.** *Journal of Materials Chemistry B* 2014, **2:**1634-1643, (DOI: 10.1039/c3tb21569e).

2. Kittler S, Greulich C, Diendorf J, Koller M, Epple M: **Toxicity of Silver Nanoparticles Increases during Storage Because of Slow Dissolution under Release of Silver Ions.** *Chemistry of Materials* 2010, **22:**4548-4554, (DOI.

3. Kreyling WG, Semmler M, Erbe F, Mayer P, Takenaka S, Schulz H, Oberdörster G, Ziesenis A: **Translocation of ultrafine insoluble iridium particles from lung epithelium to extrapulmonary organs is size dependent but very low.** *Journal of Toxicology and Environmental Health-Part A* 2002, **65:**1513-1530, (DOI.

4. Semmler M, Seitz J, Erbe F, Mayer P, Heyder J, Oberdorster G, Kreyling WG: **Long-term clearance kinetics of inhaled ultrafine insoluble iridium particles from the rat lung, including transient translocation into secondary organs.** *Inhalation Toxicology* 2004, **16:**453-459, (DOI.

5. Osier M, Oberdorster G: **Intratracheal inhalation vs intratracheal instillation: differences in particle effects.** *Fundamental and Applied Toxicology* 1997, **40:**220-227, (DOI.

6. Kreyling WG, Holzwarth U, Haberl N, Kozempel J, Wenk A, Hirn S, Schleh C, Schäffler M, Lipka J, Semmler-Behnke M, Gibson N: **Quantitative biokinetics of titanium dioxide nanoparticles after intratracheal instillation in rats: Part 3.** *Nanotoxicology* 2017, **11:**454-464, (DOI: 10.1080/17435390.2017.1306894).

7. Kreyling WG, Möller W, Holzwarth U, Hirn S, Wenk A, Schleh C, Schäffler M, Haberl N, Gibson N, Schittny JC: **Age-Dependent Rat Lung Deposition Patterns of Inhaled 20 Nanometer Gold Nanoparticles and their Quantitative Biokinetics in Adult Rats.** *ACS Nano* 2018, **12(8):**7771-7790, (DOI: 10.1021/acsnano.8b01826).

8. Hirn S, Semmler-Behnke M, Schleh C, Wenk A, Lipka J, Schaffler M, Takenaka S, Moller W, Schmid G, Simon U, Kreyling WG: **Particle size-dependent and surface charge-dependent biodistribution of gold nanoparticles after intravenous administration.** *European Journal of Pharmaceutics and Biopharmaceutics* 2011, **77:**407-416, (DOI: 10.1016/j.ejpb.2010.12.029).

9. Kreyling WG, Hirn S, Moller W, Schleh C, Wenk A, Celik G, Lipka J, Schaffler M, Haberl N, Johnston BD, et al: **Air-blood barrier translocation of tracheally instilled gold nanoparticles inversely depends on particle size.** *ACS Nano* 2014, **8:**222-233, (DOI: 10.1021/nn403256v).

10. Schleh C, Semmler-Behnke M, Lipka J, Wenk A, Hirn S, Schaffler M, Schmid G, Simon U, Kreyling WG: **Size and surface charge of gold nanoparticles determine absorption across intestinal barriers and accumulation in secondary target organs after oral administration.** *Nanotoxicology* 2012, **6:**36-46, (DOI: 10.3109/17435390.2011.552811).

11. Bolle I, Eder G, Takenaka S, Ganguly K, Karrasch S, Zeller C, Neuner M, Kreyling WG, Tsuda A, Schulz H: **Postnatal lung function in the developing rat.** *Journal of Applied Physiology* 2008, **104:**1167-1176, (DOI: 10.1152/japplphysiol.00587.2007).

12. Geiser M, Stoeger T, Casaulta M, Chen S, Semmler-Behnke M, Bolle I, Takenaka S, Kreyling WG, Schulz H: **Biokinetics of nanoparticles and susceptibility to particulate exposure in a murine model of cystic fibrosis.** *Part Fibre Toxicol* 2014, **11:**19, (DOI: 10.1186/1743-8977-11-19).

13. Oeff K, Konig A: **[Blood volume of rat organs and residual amount of blood after blood letting or irrigation; determination with radiophosphorus-labeled erythrocytes.].** *Naunyn Schmiedebergs Arch Exp Pathol Pharmakol* 1955, **226:**98-102, (DOI.

14. Lee HB, Blaufox MD: **Blood volume in the rat.** *J Nucl Med* 1985, **26:**72-76, (DOI.

15. Danscher G, Stoltenberg M: **Silver enhancement of quantum dots resulting from (1) metabolism of toxic metals in animals and humans, (2) in vivo, in vitro and immersion created zinc-sulphur/zinc-selenium nanocrystals, (3) metal ions liberated from metal implants and particles.** *Progress in Histochemistry and Cytochemistry* 2006, **41:**57-139, (DOI.

16. Loeschner K, Hadrup N, Qvortrup K, Larsen A, Gao X, Vogel U, Mortensen A, Lam HR, Larsen EH: **Distribution of silver in rats following 28 days of repeated oral exposure to silver nanoparticles or silver acetate.** *Particle and Fibre Toxicology* 2011, **8:**18, (DOI: 10.1186/1743-8977-8-18).

17. van der Zande M, Vandebriel RJ, Van Doren E, Kramer E, Herrera Rivera Z, Serrano-Rojero CS, Gremmer ER, Mast J, Peters RJ, Hollman PC, et al: **Distribution, elimination, and toxicity of silver nanoparticles and silver ions in rats after 28-day oral exposure.** *ACS Nano* 2012, **6:**7427-7442, (DOI: 10.1021/nn302649p).

18. (A.R.A.) ARA: **Multiple-path particle dosimetry model (MPPD version 3.0).** 2009, (DOI.

19. Semmler-Behnke M, Takenaka S, Fertsch S, Wenk A, Seitz J, Mayer P, Oberdorster G, Kreyling WG: **Efficient elimination of inhaled nanoparticles from the alveolar region: evidence for interstitial uptake and subsequent reentrainment onto airways epithelium.** *Environmental Health Perspectives* 2007, **115:**728-733, (DOI: 10.1289/ehp.9685).

20. Kreyling WG, Holzwarth U, Schleh C, Hirn S, Wenk A, Schäffler M, Haberl N, Semmler-Behnke M, Gibson N: **Quantitative biokinetics over a 28 day period of freshly generated, pristine, 20 nm titanium dioxide nanoparticle aerosols in healthy adult rats after a single two-hour inhalation exposure.** *Particle and Fibre Toxicology* 2019, **16:**29, (DOI: 10.1186/s12989-019-0303-7).
